# Phylogenetic analysis of condensation domains in NRPS sheds light on their functional evolution

**DOI:** 10.1186/1471-2148-7-78

**Published:** 2007-05-16

**Authors:** Christian Rausch, Ilka Hoof, Tilmann Weber, Wolfgang Wohlleben, Daniel H Huson

**Affiliations:** 1 Center for Bioinformatics Tübingen (ZBIT), Eberhard-Karls-Universität Tübingen, Sand 14, 72076 Tübingen, Germany; 2 Center for Biological Sequence Analysis, BioCentrum, Danmarks Tekniske Universitet, Building 208, 2800 Lyngby, Denmark; 3 Department of Microbiology/Biotechnology, Eberhard-Karls-Universität Tübingen, Auf der Morgenstelle 28, 72076 Tübingen, Germany

## Abstract

**Background:**

Non-ribosomal peptide synthetases (NRPSs) are large multimodular enzymes that synthesize a wide range of biologically active natural peptide compounds, of which many are pharmacologically important. Peptide bond formation is catalyzed by the Condensation (C) domain. Various functional subtypes of the C domain exist: An ^L^C_L _domain catalyzes a peptide bond between two L-amino acids, a ^D^C_L _domain links an L-amino acid to a growing peptide ending with a D-amino acid, a Starter C domain (first denominated and classified as a separate subtype here) acylates the first amino acid with a *β*-hydroxy-carboxylic acid (typically a *β*-hydroxyl fatty acid), and Heterocyclization (Cyc) domains catalyze both peptide bond formation and subsequent cyclization of cysteine, serine or threonine residues. The homologous Epimerization (E) domain flips the chirality of the last amino acid in the growing peptide; Dual E/C domains catalyze both epimerization and condensation.

**Results:**

In this paper, we report on the reconstruction of the phylogenetic relationship of NRPS C domain subtypes and analyze in detail the sequence motifs of recently discovered subtypes (Dual E/C, ^D^C_L _and Starter domains) and their characteristic sequence differences, mutually and in comparison with ^L^C_L _domains. Based on their phylogeny and the comparison of their sequence motifs, ^L^C_L _and Starter domains appear to be more closely related to each other than to other subtypes, though pronounced differences in some segments of the protein account for the unequal donor substrates (amino vs. *β*-hydroxy-carboxylic acid). Furthermore, on the basis of phylogeny and the comparison of sequence motifs, we conclude that Dual E/C and ^D^C_L _domains share a common ancestor. In the same way, the evolutionary origin of a C domain of unknown function in glycopeptide (GP) NRPSs can be determined to be an ^L^C_L _domain. In the case of two GP C domains which are most similar to ^D^C_L _but which have ^L^C_L _activity, we postulate convergent evolution.

**Conclusion:**

We systematize all C domain subtypes including the novel Starter C domain. With our results, it will be easier to decide the subtype of unknown C domains as we provide profile Hidden Markov Models (pHMMs) for the sequence motifs as well as for the entire sequences. The determined specificity conferring positions will be helpful for the mutation of one subtype into another, e.g. turning ^D^C_L _to ^L^C_L_, which can be a useful step for obtaining novel products.

## Background

**Figure 1:**
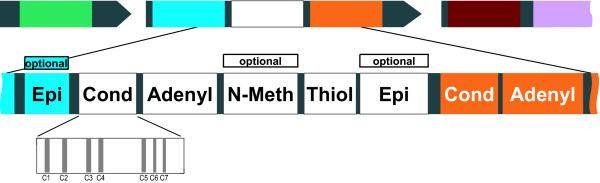
**Modular structure of NRPSs**. Module and domain structure of NRPS. Top, center: one complete NRPS consisting of three modules. Bottom: enzymatic domains contained in a complete module: *Cond: *Condensation domain (the detail shows the approximate positions of the seven motifs shown in detail in Fig. 2), *Adenyl: *Adenylation domain (A domain), *N-Meth: *N-methylation domain (optional – does not appear in all NRPS), *PCP: *Thiolation domain (T domain or Peptidyl Carrier Protein domain), *Epi: *Epimerization domain (optional). Other optional domains are: Heterocyclization, Oxidation, Reduction and Formylation domains.

The biologically active products synthesized by non-ribosomal peptide synthetases (NRPSs) are of interest for a variety of reasons: Pharmaceutically, a rich collection of them are used as drugs like antibiotics (e.g. penicillin and vancomycin), anti-tumorals and cytostatics (e.g. bleomycin), anti-inflamatorials and immunosuppressants (e.g. cyclosporin A), toxins (*α*-amanitine which is found in *Amanita phalloides *(death cap)), or siderophores. Scientifically, it is a challenge to discover how these structurally complex macromolecules are synthesized by the concerted interworking of the multi-domain proteins NRPS and polyketide synthases (PKS) that synthesize a peptide or ketide backbone with several other modifying and "decorating" enzymes (halogenases, glycosyl transferases etc.). NRPS belong to the family of megasynthetases, which are among the largest known enzymes with molecular weights of up to ~2.3 MDa (~21,000 residues) [1]. They possess several modules, each of which contains a set of enzymatic domains that, in their specificity, number, and organization, determine the primary structure of the corresponding peptide products; for a recent review on NRPS, see Sieber and Marahiel [2], and Lautru and Challis [3]. A complete module contains at least three enzymatic domains (see Fig. 1).

The adenylation (A) domain specifically recognizes one amino acid (or hydroxy acid) and activates it first through the formation of an aminoacyl adenylate and then via covalent bonding of the activated amino acid as a thioester to the 4'-phosphopantetheinyl (4'PPant) cofactor of the peptidyl carrier protein (PCP domain, also called phosphopantetheine attachment site or thiolation (T) domain). The third compulsory domain is the Condensation (C) domain, which catalyzes the elongation reaction of the peptidyl chain tethered to the phosphopantetheinyl arm of the upstream T domain to the amino acid bound to the downstream T domain (reviewed by Lautru and Challis [3]). This is why the first module of an NRPS usually does not contain a C domain, but only the second module has the domains CAT. The exceptions are C domains, which we name *Starter C *domains; these acylate the first amino acid with a fatty acid (with a *β*-hydroxy-carboxylic acid to be precise as we will discuss below). Chain elongation is terminated by the action of a thioesterase (TE) domain. It is usually the final domain of the last module in the assembly line and catalyzes either the hydrolysis or the intramolecular cyclization of the peptide chain, yielding a linear or macrocyclic product [4]. Although the multi-domain proteins NRPS and PKS are also found in fungal and plant genomes, most of the known sequences stem from bacteria. The bacterial order *Actinomycetales *is known for the wealth of secondary metabolites produced by its members and comprises, among others, *Streptomyces *species, *Corynebacteria *and *Mycobacteria*. The majority of all currently known antibiotics and other therapeutic compounds are derived from *Streptomycetes *[5]. Many members of *Corynebacteria *and *Mycobacteria *are human pathogens which produce toxins as secondary metabolites. The structural and functional diversity of non-ribosomal peptides, unlike ribosomally synthesized peptides, arises from the incorporation of unusual amino acids: During the assembly of the peptide backbone by the NRPS, both proteinogenic and non-proteinogenic amino acids (e.g. ornithine), including D-amino acids, may be integrated and modified "on-the-fly" by enzymatic domains within the NRPS protein. Possible (optional) modifications of the building blocks (= amino acids) are N-acylation of the first amino acid, epimerization (into D-amino acids), N-methylation, or cyclization of amino acids (cysteine, serine or threonine) with an amide-nitrogen of the peptide "backbone", resulting in oxazolines (e.g. in vibriobactin) and thiazolines (e.g. in bacitracin); these can be further oxidized or reduced by special domains [2], and further halogenation or hydroxylation may be mediated by specialized domains. Occasionally dehydration is performed on serines, resulting in dehydroalanine [6]. Further modifications – glycosylation or phosphorylation – are usually performed by so-called "decorating" enzymes, usually clustered in proximity to the NRPS genes on the chromosome [2].

In this paper, we report on the functional variants (subtypes) and homologues of the Condensation (C) domain of NRPS. All C domain sequences of this study were extracted from NRPS that were detected in all available completely sequenced bacterial genomes and a comprehensive collection of annotated biosynthesis clusters. Besides A domains (and thioesterase II domains; see Sieber and Marahiel [2]) C domains also show specificity for their substrates (see below). An in-depth understanding of their function is thus crucial for re-engineering NRPS to produce novel bioactive compounds. In practice, it has been shown that it is possible to engineer synthetic systems for the production of novel products: Stachelhaus *et al*. [7] demonstrated that domain swapping, which is the recombination of domain-coding regions of desired specificity to a synthetic fusion protein, worked to create new variants of surfactin and is thus one possibility, although only one amino acid position in the product was varied, which did not alter its activity, and the total yield was very low (0.5 % of wilt-type yield).

Because C domains have been shown to have non-negligible specificity for the amino acid that is activated by the downstream A domain, swapping whole modules or insertion/deletion seems to be more promising, provided that the integrity of the functional domains is carefully maintained and the modules are dissected in their linker regions [8,9]. Nevertheless, reduced catalytic efficiency and product yield is a serious problem. A less invasive strategy involves the manipulation of the domains' specificity by point mutations as demonstrated by Eppelmann *et al*. [10] for the A domain. Therefore, an in-depth knowledge of all functional subtypes and homologues of the C domains is indispensable. In this report, we reconstruct their phylogeny and reveal the sequence motifs of all subtypes and homologues, and their mutual differences. The insights gained will be helpful in future attempts to turn one sub-specificity into another, e.g. changing the stereoselectivity of the C domain.

Furthermore, we have analyzed C domains and Epimerization (E) domains of glycopeptide NRPS. In these proteins, two Condensation domains preceded by former (now inactive) Epimerization domains have gained opposite stereoselectivity, probably due to convergent evolution, for which we accumulate evidence. Additionally, we discuss the origin of a C domain (often referred to as X* domain) at the C-terminus of glycopeptide NRPS, which is thought to be inactive.

## Results and Discussion

### Current knowledge of subtypes ^L^C_L_, ^D^C_L_, Cyc, and Dual E/C

The C domain has two binding sites: one for the electrophilic donor substrate (the acyl group of the growing chain) and one for the nucleophilic acceptor substrate (the activated amino acid). The condensation reaction involves catalysis of a nucleophilic attack by the amino group of the aminoacyl adenylate bound to the downstream PCP on the acyl group of the growing peptide chain which is bound to the upstream PCP [2,11]. The acceptor site of the C domain was shown to exhibit a strong stereoselectivity and significant side chain selectivity. The selectivity towards a specific side chain seems to be less pronounced at the donor site which, however, exhibits strong stereoselectivity [3].

In particular, C domains succeeding an E domain are expected to show specificity towards the configuration (L or D) of the C-terminal residue that is bound at the donor site because the preceding E domain does not specifically catalyze the epimerization from L to D but provides a mixture of configurations. It is the role of the C domain to select the correct enantiomer [11]. Moreover, the C domain represents some kind of selectivity filter in that it supports the selection of the correct downstream nucleophile and prevents product mixtures [2].

C domains immediately downstream of E domains were shown to be D-specific for the upstream donor and L-specific for the downstream acceptor, thus catalyzing the condensation reaction between a D- and an L-residue. These C domains were termed ^D^C_L_-catalysts because of this behavior [12].

**Figure 2:**
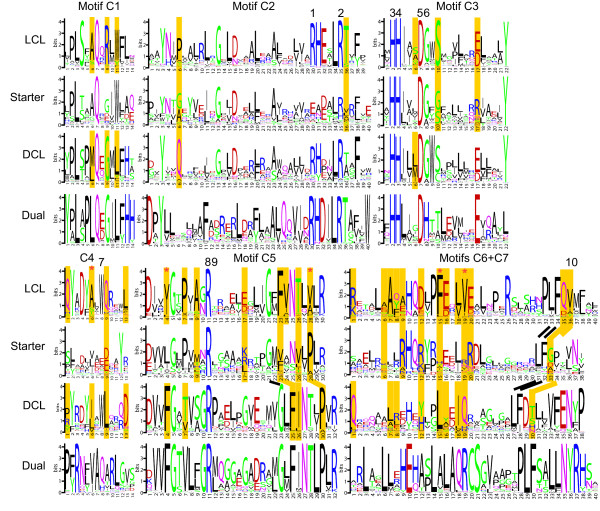
**Core motifs C1 through C7 of C domain subtypes ^L^C_L_, Starter, ^D^C_L _and Dual E/C domains**. Compared to Marahiel *et al*. [29], motifs are extended in both directions to include more significantly conserved positions. Yellow bars indicate significant specificity determining positions between ^L^C_L_, Starter and ^D^C_L _domains; those with red stars on top are the most significant positions. Numbers above the letter stacks indicate residues of functional and structural importance refered to in Subsection "Key residues in Condensation domains" and Table 1.

Accordingly, ^L^C_L_-catalysts promote the condensation of two L-amino acids. Both ^L^C_L_- and ^D^C_L_-catalysts possess a conserved His-motif in their active site. The consensus sequence of this motif is HHxxxDG where x denotes any residue (see Fig. 2, motif 3). The second His-residue seems to be essential for the catalytic function of the domain [2].

As a third type of C domain, so-called Dual Epimerization/Condensation (E/C) domains have recently been identified. This finding was based on the observation of NRPS which had products that contained D-residues although the NRPS itself did not show an E domain in the corresponding module. Biochemical experiments supported the hypothesis that Dual E/C domains exist which are ^D^C_L_-catalysts with epimerase activity [13]. In the assembly line, a Dual E/C domain follows directly after a C-A-T module which activates and incorporates an L-amino acid. The module which contains the Dual domain also activates an L-amino acid. Then the Dual domain catalyzes the epimerization of the L-residue into D configuration and subsequently promotes the condensation of those two residues. In addition to the active site His-motif which is found in all C domains, Dual E/C domains exhibit a second His-motif, HH[I/L]xxxxGD, which is located close to the N-terminus of the domain [13] (It is partly located on motifs C1 & C2; see Fig. 2.)

C domains may be replaced by Heterocyclization (Cyc) domains which catalyze both peptide bond formation and subsequent cyclization of cysteine (Cys), serine (Ser), and threonine (Thr) residues. The five-membered heterocyclic rings which result from this reaction are important for chelating metals or interaction with proteins, DNA or RNA. Cyc domains are structurally related to C domains and are supposed to be evolutionary specialized C domains [2]. In Cyc domains, however, the active site His motif is replaced by another conserved motif, DxxxxD. Keating *et al*. [14] found that the aspartate (Asp, D) residues are critical for both condensation and heterocyclization.

### Collected C domain sequence data and their phylogenetic tree

A total of 481 Condensation domains (including their homologues, Epimerization and Heterocyclization domains) were extracted from 182 (non-identical) NRPS and 31 NRPS/PKS hybrid sequences found in 62 bacterial genomes out of the 256 bacterial genomes screened, employing pHMMs as described in Section Methods (Note that only one genome was considered for our analysis if sequences of several strains of the same species were available, which reduced the number of NRPS or 'hybrid NRPS/PKS' containing genomes from 62 to 43). Altogether 108 C domains were obtained from 42 NRPS sequences from gene clusters downloaded from the UniProt database. After removing doublets, all 525 non-identical C domains and homologues obtained were multiply aligned and phylogenetic trees were built. The resulting tree topology was clearly dominated by the functional categories that are known for C domains (as described in the previous section), rather than species phylogeny or substrate specificity alone. The four main functions are: *1*. condensation performed by ordinary C domains; *2*. condensation and subsequent heterocyclization catalyzed by Heterocyclization (Cyc) domains; *3*. epimerization followed by condensation which are both catalyzed by a Dual E/C domain; *4*. Starter domains (see below) which are found on initiation (= first) modules and acylate the subsequent amino acid.

**Figure 3:**
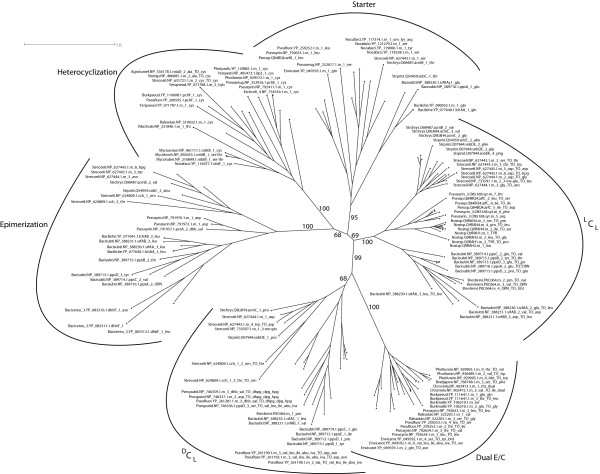
**Phylogenetic trees of all C subtypes**. Phylogenetic tree of all C subtypes (^L^C_L_, ^D^C_L_, Starter, Dual E/C, Epimerization and Heterocyclization domains). The phylogeny was reconstructed using phyml, employing the JTT model of amino acid substitution and a gamma-distributed rate variation with four categories. The support values are based on 100-fold bootstrapping.

Ordinary C domains may further be classified into ^L^C_L_-catalysts and ^D^C_L_-catalysts according to the stereochemistry of their substrates. The existence of all these functional subtypes is reflected by the phylogeny. Fig. 3 shows a phylogenetic tree for subsets of each C domain subtype, as the whole tree of 525 taxa is far too large to be displayed here (see Additional files 1 and 2). The tree of all taxa showed a similar topology perfectly reflecting the functional categories.

For further analysis, the different subtypes were examined separately. While Cyc and Dual E/C domains could be identified by means of their characteristic sequence motifs (see Section Methods/Predicting of functional subtypes), ^L^C_L_- and ^D^C_L_-catalysts were either distinguished according to their domain structure or by their position in the phylogenetic tree. By this, 275 domains of all 525 C domains were classified as being ^L^C_L_-catalysts, 69 were ^D^C_L_-catalysts and 42 were Starter C domains (see next section).

### Description of a new C domain subtype: The Starter C domain

When analyzing the Condensation (C) domain phylogeny, it became apparent that some domains did not cluster with the known C domain subtypes. A closer look at the location of these deviating C domains revealed that all of them were the very first C domain of the corresponding NRPS assembly line. The remaining C domains of these assembly lines appeared in other subtrees in the phylogeny.

Included in this set of starter C domains are those stemming from the biosynthesis clusters for the lipopeptides surfactin [15], lichenysin [16], fengycin [17] and arthrofactin [18]. These lipopeptides are characterized by a *β*-hydroxyl fatty acid which is connected to the first amino acid of the peptide chain [19]. The peptide synthetases involved in the production of these lipopeptides all have a C domain as their very first domain. This C domain is supposed to serve as an acceptor for a fatty acid which is transferred from an acyltransferase [19]. This acylation process has also been observed for surfactin [20] and fengycin biosynthesis [21]. Moreover, common to the Starter C domains of these biosynthesis clusters is their low sequence similarity to the remaining C domains of the same biosynthesis cluster [19].

The same has been observed for the synthesis of the acidic lipopeptide CDA in *Streptomyces coelicolor *[22] and the recently identified lipopeptide produced by protein NP_960354.1 of *Mycobacterium avium *[23]. The Starter C domain of the pristinamycin cluster appears to diverge from this pattern at the first view. The C domain is the first domain of the polypeptide SnbC but the biosynthesis of pristinamycin is initiated by SnbA, which contains an A domain that activates 3-hydroxypicolinic acid (3-hydroxypyridine-2-carboxylic acid, "2-hydroxy-6-azabenzoate") but lacks an ACP [24]. SnbA is homologous to EntE, which contains an A domain specific for 2,3-dihydroxybenzoate (DHB) and which is involved in the biosynthesis of enterobactin [25]. A similar organization can be found in actinomycin biosynthesis. The process is initiated by AcmA, which activates 4-methyl-3-hydroxyanthranilic acid (MHA, 4-methyl-3-hydroxy-2-aminobenzoate) [26]. In conclusion, what the C domains of SnbC, AcmB and EntF have in common is that they catalyze bond formation between a derivative of salicylic acid (2-hydroxy-benzoate) and an *α*-amino acid. Assured by the fact that these Starter C domains match significantly well to the profile HMM built from the Starter C domain sequences that process *β*-hydroxy fatty acids, we compared salicylic acid with *β*-hydroxy fatty acids. Because both are *β*-hydroxy-carboxylic acids with no amino-substituent at the *α *position, as *a*-amino acids would have, we assume that this is the structural characteristic recognized by the prototype of Starter C domains. The profile HMM built from all Starter C domains in our data set (together with the pHMMs of the other domains) presents a powerful instrument for exploring and understanding tricky NRPS domain-product relations.

Note that Formylation domains as found, for example at the N-terminus of linear gramicidin synthetase subunit A [27] are not C domains but belong to the Pfam "formyl transferase" domain family.

### Characteristic Sequence Motifs of ^L^C_L_, ^D^C_L_, Starter C domains and Dual E/C domains

The different core motifs in Condensation domains have first been described by de Crécy-Lagard *et al*. [28] and recompiled by Marahiel *et al*. [29] but have never been updated since then. The core motifs of the C domain homologues, Epimerization and Heterocyclization domain are listed in the publication by Marahiel *et al*. [29] but the sequence motifs of the recently discovered ^D^C_L _domains [12,30] as well as the Dual E/C [13] domains have never been comprehensively analyzed. Moreover the Starter C domain has not yet been recognized in the literature as a proper separate subtype.

The sequence motifs represented in Fig. 2 improve the C domain core motif consensus sequences published by Marahiel *et al*. [29] which, at that time, were based on much fewer sequences and did not differentiate between the C domain subtypes. The motifs are represented as sequence logos [31] which make it easier to identify variably conserved positions compared to simple consensus sequences. We adhere to the core motifs identified by Marahiel *et al*. [29], and also show the surrounding "landscape" if there are highly conserved positions nearby, especially if they are important for distinguishing between the C domain subtypes. The motifs were built on the basis of 40 verified and 198 predicted ^L^C_L _sequences, in which "predicted" means that they were classified based purely on their position in the phylogenetic tree while "verified" sequences were checked individually taking into account their position in the succession of neighboring NRPS domains, the presence of discriminative unique motifs (see Methods Section) and/or literature information. For the ^D^C_L _motifs, 23 verified and 46 predicted sequences were used, 7 verified and 35 predicted for the Starter domains, and domains 9 verified and 47 predicted for the Dual E/C domains.

### Key residues in Condensation domains derived from the literature

Based on three publications, four residues are likely to be essential for the catalytic activity of the C domain. The most important residue is the 2nd His of the active site His-motif [32].

Furthermore, six residues have been identified as being structurally important or as playing a role in correct folding of the domain. In the following, these residues are presented, grouped by their role (the numbering is according to their linear occurrence on the peptide; see Fig. 2). This information is also presented in Table 1 where the sites are sorted by their relative position in the domain.

**Table 1:** Residues of importance for catalytic activity, structure or correct folding. Residues for which the importance has been previously determined are shown in Fig. 2, giving their numbers, their role and the bibliographic reference of the appropriate mutation study.

Nb. in Fig. 2	Importance:	Position is homologous to:	Reference:
1	structure	Arg62 (R) in TycB1	[34]
2	folding	Arg67 (R) in TycB1	[34]
3	folding	His146 in TycB1 (1st His of active site His-motif)	[34]
4	catalytic activity	His126 (2nd His of the active site His-motif) in VibH	[14,33,34]
5	structure	Asp130 (D) in VibH	[14,33,34]
6	catalytic activity	Gly131 (G of the active site His-motif) in VibH	[33]
7	folding	Trp202 (W) in TycB1	[34]
8	structure	Arg263 (R) in VibH = Arg278 (R) in EntF	[14,33]
9	catalytic activity	Trp264 (W) in VibH according to Keating *et al*., but absent in ^L^C_L_, ^D^C_L _and Starter C domains	[14]
10	catalytic activity	Asn335 (N) in VibH	[33]

#### Residues of importance for catalytic activity of the domain

#4 His 126 (2nd His of the active site His-motif) with respect to (w.r.t.) VibH [14,33,34]

#9 Trp264 (W) is catalytically important in VibH according to Keating *et al*. [14], but the corresponding position is not conserved in any of the C domain subtypes ^L^C_L_, ^D^C_L _or Starter.

#10 Asn335 (N) w.r.t. VibH [33]

#6 Gly131 (G of the active site His-motif) w.r.t. VibH [33]

#### Residues of structural importance

#1 Arg62 (R) w.r.t. TycB1 [34]

#5 Asp130 (D) w.r.t. VibH [14,33,34]

#8 Arg263 (R) w.r.t. VibH [14] = Arg278 (R) w.r.t. EntF [33]

#### Residues important for correct folding

#2 Arg67 (R) w.r.t. TycB1 [34]

#3 His146 w.r.t. TycB1 (1st His of active site His-motif) [34]

#7 Trp202 (W) w.r.t. TycB1 [34]

#### ^L^C_L _vs. ^D^C_L_

^L^C_L _and ^D^C_L _domains do not differ significantly in any of the residues identified as being of catalytic or structural importance (except residues Nb. 9 and Nb. 10). However, using methods described in Section Methods, 20 positions in which ^L^C_L _and ^D^C_L _have significant differences according to SDPpred [35] could be detected, plus 5 additional high scoring positions within the extended motifs according to FRpred [36]. When comparing the different motifs, motif C4 differs noticeably between ^L^C_L _and ^D^C_L _subtypes. The same is true for the region downstream of C4 (after the mutually very conserved TRP at pos. 184 in VibH coordinates) where a moderately conserved motif LPxDxxRP is seen in ^L^C_L _which is completely absent in ^D^C_L _(see Additional file 3).

### ^L^C_L _vs. Starter domain

While not being conserved at residues Nb. 5, Nb. 7, Nb. 9, and Nb. 10, all remaining 6 functionally important residues are highly conserved throughout the putative Starter domains. When comparing ^L^C_L _and Starter domains, 18 discriminative positions were found by SDPpred and 5 more were found in the motifs by FRpred. Those positions are highlighted in Fig. 2. Common to these residues is the fact that they seem to be highly conserved among extender (= ^L^C_L_) domains but show no conservation among Starter C domains. When we compare C domain sequence motifs, it is apparent that motifs C2 and C4, despite being well conserved in ^L^C_L_, are unconserved in Starter domains, which presumably can be explained by the much broader structural range of substrates processed by Starter domains.

### What the phylogeny tells about the relationship of ^L^C_L _vs. Starter and ^D^C_L _vs. Dual E/C domains

**Figure 4:**
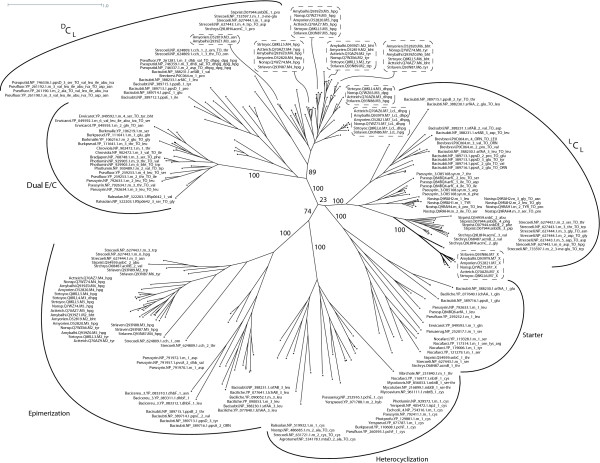
**Phylogenetic trees of all C subtypes including C domains from glycopeptide clusters**. Additionally, this tree includes all C domains of glycopeptide antibiotic biosynthesis clusters (in dashed boxes). The phylogeny was reconstructed using phyml, employing the JTT model of amino acid substitution and a gamma-distributed rate variation with four categories. The support values are based on 100-fold bootstrapping.

The reconstructed phylogeny of C domain subtypes reveals that ^L^C_L _and Starter C domains are more closely related to each other than to other subtypes (see Fig. 3). Comparing sequence motifs confirms this observation, though pronounced differences in some segments of the protein (especially in motifs C2 and C3, as can be seen in Fig. 2) account for the unequal donor substrates (amino vs. *β*-hydroxy-carboxylic acid). Furthermore the phylogenetic tree shows that Dual E/C and ^D^C_L _domains share a common ancestor. We tested the reliability of the phylogenies depicted in Fig. 3 and Fig. 4 by repeating the reconstruction on biased profile alignments. These biased alignments were generated by producing MUSCLE profile-profile alignments in a step-wise manner, assuming evolutionary relationships of the different domain subtypes that are contradictory to what the original trees suggest. The topology of the resulting trees supports the shared ancestry of ^L^C_L _and Starter C domains as well as of Dual E/C and ^D^C_L _domains. In addition, we generated an alignment using DIALIGN [37], which is a non-progressive alignment method, and subsequently reconstructed a PHYML-tree based on this alignment. Here also, the Dual E/C and ^D^C_L _domains are grouped together as are ^L^C_L _and Starter C domains.

Especially in motif C5, Dual E/C and ^D^C_L _domains are very similar to each other and dissimilar to ^L^C_L _and Starter domains. This observation of the relationship between the four subtypes is consistent with the stereochemistry of the substrates, bearing in mind that Dual E/C domains function as ^D^C_L _because the substrate L-amino acid is first epimerized by the intrinsic epimerization activity of the domain [13].

Within the subtrees of ^D^C_L _and ^L^C_L _domains, the tree topology reflects the species phylogeny of the bacteria rather than substrate specificity of any kind. We analyzed this by reconstructing phylogenies for ^D^C_L _domains and ^L^C_L _domains separately to be able to see the topology within these subtypes in more detail (data not shown). The reconstructed phylogenies did not give any evidence that would support the hypothesis that C domains cluster according to their specificity towards the condensated amino acids. This analysis, however, is based on the complete C domain sequence. A strategy to investigate whether C domains exhibit substrate specificity would involve predicting putative specificity determining positions using entropy and/or conservation based approaches (e.g. SDPpred, FRpred), or inferring of putative active site residues by homology with the VibH structure (as done by Rausch et al. [38] for the adenylation domain).

### Enigmatic Glycopeptide antibiotic NRPS

Glycopeptide antibiotics are a subgroup of nonribosomal peptide antibiotics of which the best known representatives are probably vancomycin and teicoplanin. To date, all identified glycopeptide antibiotics are produced by actinomycetes. They interrupt cell wall formation of gram-positive bacteria by binding to the D-Ala-D-Ala termini of the growing peptidoglycan, thereby inhibiting the transpeptidation reaction. All glycopeptide antibiotics consist of a heptapeptide backbone which is synthesized by NRPS.

**Figure 5:**
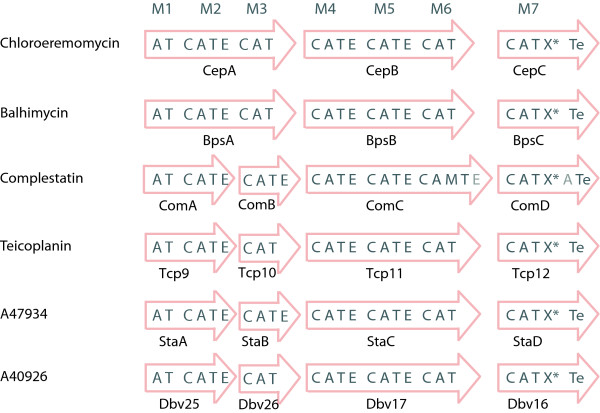
**Modular organization of NRPS involved in glycopeptide synthesis**. Domains marked in light gray (Completstatin) are inactive and corrupt. Moreover, E domains in ComB and StaB are also thought to be inactive.

Modification reactions involve extensive cross-linking of the aromatic side chains to rigidify the molecule [39,40]. The modular organization of some NRPS which were identified in glycopeptide-producing actinomycetes are depicted in Fig. 5.

All these NRPSs comprise seven modules. They show an identical domain composition, with the exceptions of module M3 in the A47934 (*sta*) and M3 and M6 in complestatin (*com*) clusters which contain an E domain not present in the other clusters. The M3-E domain, however, is assumed to be inactive [41], while the presence of an E domain in *com *M6 has not been reported elsewhere so far. We were able to detect it with an hmmpfam scan using the specific E domain pHMM. All six NRPSs contain a domain X* of unknown function. Until now, it has been characterized as an atypical C or E domain but its role in glycopeptide synthesis remains to be clarified. In general, it is assumed that the stereochemistry of a NRPS product can be predicted from its domain structure. In the case of the known glycopeptides, the domain organization implies the stereochemistry NH_2_-L-D-L-D-D-L-L-COOH, provided that the E in module M3 is inactive and that the X* domain does not function as an E domain. This stereochemistry is inconsistent with the chemically determined structure of the products: NH_2_-D-D-L-D-D-L-L-COOH [41]. The assumption is that the A domain of the first module activates a D-amino acid. For the *cep *cluster, however, Trauger and Walsh [42] show that the A domain of M1 prefers L-Leu over D-Leu in a 6:1 ratio; but on the other hand, they could not show which stereoisomer is processed further. This suggests the existence of an unknown E domain that acts on the L-Leu activated by M1. With the discovery of Dual E/C domains, a new possible strategy arises for the incorporation of a D-residue by the first module. However, no Dual E/C domain could be detected in all glyco-NRPS. Alternatively, one could imagine an external racemase as is found in the cyclosporin cluster [43], which provides a D-Leu that can be incorporated directly.

Having gained knowledge about the differences between ^L^C_L_, Starter and ^D^C_L _domains as described above, we examined all glyco-NRPSs. When we reconstructed the phylogeny of C domains including all homologous domains from glyco-NRPSs, it was staggering to find that all C domains were clustered in the ^D^C_L _subtree and the X* domain clustered in the ^L^C_L _subtree (see Fig. 4). This finding could be confirmed by analyzing all instances of the C domain motifs found in these domains. How could this be interpreted, given the fact that M4 and M7 C domains clearly act as ^L^C_L _domains, as we can tell by the stereochemistry of the products? Our hypothesis is that those C domains are former ^D^C_L _domains that have developed ^L^C_L _activity by convergent evolution. Accumulating supportive evidence is possible: When we look at the phylogeny of the C domains, the sequences of the *com *cluster from *Streptomyces lavendulae *are always most distant from the others and more closely related to the hypothetical common ancestor, implying that they can serve as a model for the archetype of glyco-C domains. It is likely that in the archetype, all C domains were true ^D^C_L _catalysts, supposing that the E domains which are still present in *com *modules M4 and M7 were still active.

In a similar way, we can trace back the origin of the X* domain: in the *com *cluster (and only there) it is followed by remnants of an adenylation domain (which has several larger insertions and deletions; see Additional file 4). This tells us that the X* domain used to be the first domain of a new module followed by an adenylation domain.

The assumption that the diverged C domains of modules M4 and M7 would have adopted mutations at positions that we have previously determined as "specificity determining positions" was disproved. Probably, a few spontaneous mutations in the ^D^C_L _domains relaxed the stereo-selectivity; supposing that this altered stereochemistry of the product resulted in a highly selective advantage (arising from a vancomycin-like product), the loss of the functional E domains in M3 and M6 would have been a selective gain. Comparing all M4 and/or all M7 C domains with all ^D^C_L _domains using SDPpred did not reveal any significant positions; comparing them against the other glyco-C domains gave thirty positions. As all glyco-C domains are very closely related and differences between them might also reflect substrate selectivity (not only stereo-selectivity) or different inter-domain interacting residues, we cannot decide which of them confer the altered stereo-selectivity. One point to notice however, is a (positively charged) His in all M4 glyco-C domains at position 6 in the extended motif C2 where an (uncharged polar) Gln is highly conserved in other ^D^C_L _domains. This position has also been selected by FRpred as a significant (= subtyping) position. The other positions do not represent mutations in highly conserved residues (data not shown). It would be necessary to check their significance experimentally with mutation studies. It would also be helpful to compare the peculiar sequences with more glyco-C domains, but others are -unfortunately – not publicly available.

However, although we could not discover which altered positions are responsible for the functional shift from ^D^C_L _to ^L^C_L _in glyco-C domains, interesting experimental questions can be formulated based on our findings. For example, one could think of mutational studies with the goal of altering the stereo-selectivity of a ^D^C_L _domain and to determine the relevant residues experimentally. A starting point could be, for example the M6 C domain of any glyco-NRPS.

### Glycopeptide-AB module M7 vs ^L^C_L_

The second His of the His-motif in motif C3 which is important for catalysis is replaced by Arg (R). Also, the Gly of the His-motif is not present but replaced by Arg in all but one X* domain. Note, however, that while the second active site His is invariant in C domains, Gly138 is not.

SDPpred predicted 13 specificity determining residues when comparing M7-X* to ^L^C_L_-domains of *Streptomyces *species. Only three of these coincide with residues of functional importance: His126, Arg278 and Asn335. Furthermore, a C terminal region could be detected in which M7-X* and ^L^*C*_L _differ strikingly. The concordance of M7-X* with the most highly conserved residues of Streptomycete ^L^C_L _domains supports the phylogenetically based suggestion that M7-X* is an inactive ^L^C_L _domain.

## Conclusion

In this study, we present the evolutionary relationship of homologues of the NRPS Condensation domain which include enzymatic domains catalyzing Epimerization, Heterocyclization, Condensation and Epimerization with subsequent Condensation in one domain (called the Dual E/C domain). The Condensation domain itself appears in three subtypes according to the stereo-chemistry of the substrates catalyzed: ^L^C_L _domains, which condense two L-aminoacids, ^D^C_L _domains, which condense a D-amino acid (N-terminal part of the growing peptide) with an L-amino acid, and Starter C domains (an expression that we coin here) which connect a *β*-hydroxy-carboxylic acid (e.g. *β*-hydroxyl fatty acid) with an L-amino acid. The phylogeny of C domain homologues is reconstructed using NRPS sequences (including hybrid NRPS) from completely sequenced genomes (43 genomes contained NRPS) and selected biosynthesis clusters, involving 525 non-identical C domain sequences. The sequence motifs of ^L^C_L_, ^D^C_L _and Starter domains have been extracted and are presented as sequence logos: for ^L^C_L _domains, this represents an update of consensus sequences published by Marahiel *et al*. [29]; ^D^C_L _and Starter domain motifs are analyzed and mutually compared for the first time. For comparison, the homologous motifs are also presented for Dual E/C domains, which were first described by *Balibar et al*. [13].

We have investigated the "mysterious" evolutionary origin of C domains in glycopeptide antibiotic synthesis clusters and have discovered that two of the six C domains present in these glyco-NRPSs appear in the ^D^C_L _subtree of the phylogenetic tree and show all DC_L _sequence motifs, although they clearly have ^L^C_L _activity. This suggests that they might be an example of convergent evolution. Even though this is probably a rare event, its possibility has to be kept in mind when uncharacterized C domains are to be classified, e.g. using profile HMMs provided as Additional files 5, 6, 7. Furthermore, we found that a C domain-like segment of glyco-NRPS, called X*, is related to the ^L^C_L _domains and is followed by remnants of an A domain, implying an additional complete module in the ancestor of glyco-NRPS.

Roongsawang *et al*. [44] have already performed a study of the phylogeny of C domains which compares the three C domain subtypes. However, this study shows no awareness of the Dual E/C domain, which has since been discovered. Moreover, we used a much more comprehensive dataset of C domain subsequences (525, as opposed to Roongasawang et al.'s 162) compiled from all complete bacterial genomes and biosynthesis clusters. Because of the omission of Dual E/C domains, their conclusions need to be revised, as we have shown.

## Methods

### Genomes and sequences

The protein sequences and GenBank entries for all completely sequenced bacterial genomes available to date were obtained from the NCBI FTP site . In total, the genomes of 256 bacterial species were downloaded and screened for NRPS protein sequences (including NRPS/PKS hybrids). Additional protein sequences of PKS and NRPS which are part of known secondary metabolite biosynthesis clusters were obtained from the UniProt database [45]. NRPSs were retrieved from 14 known biosynthesis clusters, of which 13 came from *Actinomycetes *and one from *Pseudomonas *(see Additional file 8).

### Identification of enzymatic domains

A common strategy for the identification of a specific type of domain is to use Profile Hidden Markov Models (pHMMs), which are statistical models extracted from multiple sequence alignments. In contrast to simple sequence motifs of fixed length, i.e. position specific scoring matrices, pHMMs are suited for identifying motifs that are interrupted by segments of variable length, and are used to characterize position-specific sequence similarities within a family of proteins. A collection of pHMMs for a wide array of domains and domain families is availabe from the database Pfam [46] and TIGRFAMs [47]. The pHMM implementation HMMER [48,49] and self-written Perl [50] scripts and BioPerl [51] scripts were used to search for NRPS in the genome sequences and biosynthesis clusters and to extract single domains from a given protein sequence. To identify a protein sequence as an NRPS, the occurrence of at least one complete NRPS module with one C domain, one A domain and T domain was required (Pfam accession numbers PF00668, PF00501 and PF00550), with an E-value threshold of 0.1 (thus we accepted to miss freestanding starter modules containing only A and T domains, or had to add them manually, as in the case of the biosynthesis clusters).

The Pfam pHMM Condensation (PF00668) recognizes both the Condensation (C) and Epimerization (E) domain of NRPS. The intention, however, is to be able to distinguish between these two domain types. Therefore C domain and E domain specific pHMMs were generated from a multiple sequence alignment (MSA) of Epimerization domains and non-Epimerization domains, both of which were recognized by the Pfam C pHMM. To obtain a set of Epimerization domains, all NRPS sequences with complete modules were extracted from all bacterial protein sequences in the Uniprot database [45] as described above. Whenever two consecutive C domains followed by an A domain were detected with Pfam pHMMs, the "first C" domain was extracted. That way, we obtained a set consisting mainly of E domains (151 of 237 sequences). By phylogenetic subtyping (as described below) we determined the E domain sequences from the phylogenetic tree of the "first C" domains, which were forming a distinct subtree. The E and non-E sequences were aligned with MUSCLE [52,53], and specific pHMMs were build for them with hmmbuild and hmmcalibrate from the HMMER package (As a control, it was not possible to detect E domains in the 771 "second C" domains). The domain sequence covered by our own pHMMs for C and E domains is identical with that of the Pfam Condensation pHMM; in other words it extends from four positions before our extended C1 motif to the fourth position after the extended C5 motif (these motifs were first revealed by de Crécy-Lagard *et al*. [28] and reviewed by Marahiel *et al*. [29]). Phylogenetic reconstruction is always based on this part of the C domain (see Fig. 2). To extract the complete N-terminal part of the C domains, we followed the dissections applied by Roche and Walsh [33] and checked the secondary structure with Quick2D of the MPI Bioinformatics Toolkit [54,55].

### Generation of multiple sequence alignments

The quality of a reconstructed phylogenetic tree crucially depends on the underlying multiple sequence alignment. All sequence alignments in our study were generated using MUSCLE [52,53]. The alignment algorithm can be divided into three stages. First, a progressive alignment is built based on a UPGMA guide-tree. In the second stage, the underlying guide-tree is iteratively improved, yielding a new progressive alignment. The third stage involves refinement of the tree: Based on the tree, bipartitions of the dataset are produced; their profiles are extracted and realigned to each other. Thus, the finally generated alignment is not solely based on a single guide-tree, which is why we can rule out that the phylogenies reconstructed on the basis of these alignments merely reflect the guide-tree used in the first step of the algorithm.

### Predicting substrate specificity

C domains catalyze the condensation of two amino acids, thus, they have two binding sites: the acceptor and the donor site. To be able to investigate whether the substrate specificity of one of these sites influences the phylogeny of the domain, the specificity of the preceding and succeeding A domain in the assembly line was predicted with the NRPSpredictor [38] and stored for each C domain.

### Predicting functional subtypes

Functional subtypes may be distinguished on the basis of sequence features, domain architecture or clustering behavior during tree reconstruction. Condensation and Heterocyclization domains may be discriminated by the sequence motif they exhibit at their active site. The occurrence of a sequence motif within a longer sequence can be detected with the help of a position specific score matrix (PSSM) [48]. PSSMs were generated and applied for the detection of the active site His-motif of the C domain and the DxxxxD-motif of the Heterocyclization domain. These were used to discriminate between the two subtypes. The His-motif was built from 86 sequences and the Cyc motif from 15 sequences. The PSSMs were only applied to a region of 100 residues which was expected to contain the active site. In addition, a PSSM was generated for the N-terminal His-motif found in Dual E/C domains. It was constructed from 55 sequences which had been identified as Dual E/C domains by their clustering behavior in the phylogeny and by additional visual inspection of the alignment. The PSSM was applied for validation purposes to make sure that this N-terminal His-motif is unique to Dual E/C domains and cannot be found in any other C domain subtype. Predicting whether a C domain is a ^L^C_L_- or a ^D^C_L_-catalyst was established according to the observed domain organization of the modules in an NRPS sequence (^D^C_L_-catalysts were first described by Luo *et al*. [30]). It is assumed that the role of a module with the domain structure C-A-T-E is the activation and epimerization of a residue that is in the L stereo configuration with the intention of incorporating a D residue into the final product. Alongside this, a C domain directly following an E domain is expected to be selective for residues in D-configuration, which is why it was assigned to the ^D^C_L_-type. All other C domains were assumed to be ^L^C_L_-catalysts. Classification as a ^D^C_L_-catalyst is supposed to be fairly reliable. A false positive should only occur if the preceding epimerase turns out to be nonfunctional. The ^L^C_L _classification, however, is prone to errors when the respective C domain is the very first (N-terminal) domain in the protein. In this case, the type of the condensation reaction can only be assigned if the order in which the proteins act in the assembly line is known. To overcome this problem, we checked all assignments with the classification suggested by the phylogeny.

If the order of the subunits is unknown, temporarily incorrect assignments can only be revised later in the analysis.

### Analysis of multiple sequence alignments for specificity determining positions

In a set of homologous enzymes, we may find subsets that each contain sequences with one distinct substrate specificity. These subsets of common function are called subtypes and often vary at certain positions, whereas the same positions may be conserved within a given subtype. Li *et al*. [56] call these specificity-determining residues (SDR); Kalinina *et al*. [35] refer to them as specificity determining positions (SDP). To determine SDPs from an alignment, calculating each column's mutual information is a possible way, as described by Li *et al*. [56] and Kalinina *et al*. [35]. For this paper, SDPs were determined using the freely accessible SDPpred server [35]. Here, the mutual information is based on so-called smoothed frequencies, which allow substitution of residues with similar physico-chemical properties. In addition to that, the significance of the mutual information of each position is estimated by calculating Z-scores and evaluating their significance. Predictions by SDPpred were compared with the highest scoring positions predicted by FRpred [36,57] which combines a mutual information term with a conservation score.

### Reconstruction of phylogenetic trees

Several methods were applied for reconstructing phylogenetic trees from the multiple sequence alignments that were generated for each domain type. Trees presented in this article were reconstructed using protein sequences, as amino acid sequences are preferred to nucleotide sequences because they are more conserved and are not influenced by compositional bias like G+C content and codon usage. In addition, the mathematical model for the evolutionary change of amino acid sequences is much simpler than that of nucleotide sequences, which reduces the risk that the phylogeny is based on wrong evolutionary assumptions, since just a suitable substitution matrix has to be selected [58]. The amino acid substitution matrix employed in this study was the Jones-Taylor-Thornton (JTT) matrix [59].

In some cases, the rate of amino acid substitution may be assumed to be the same for all positions in the alignment. In general, however, this does not reflect reality since the substitution rate is usually higher at positions of lower functional importance. A more realistic model is achieved if the substitution rate is taken to vary among sites according to the gamma distribution [60].

Apart from PHYLIP [61], all methods used in this study offer an estimation of parameter *α *which determines the shape of the Γ distribution as an option. Whenever a gamma distributed rate variation was assumed, four gamma-rate categories were used to approximate the distribution. Several tree reconstruction methods were applied to each dataset to determine whether different methods yield different topologies, which in turn would indicate that the proposed topologies are unreliable. As a distance-based method, the Neighbor-Joining (NJ) method [62] was applied. The distances were calculated with the program protdist and NJ was performed with neighbor, both available from the PHYLIP package. For NJ, only uniform substitution rates were used. As a maximum likelihood method, the programs IQPNNI [63] and PHYML [64] were applied.

Bootstrapping [65] was performed to test the reliability of the topologies.

In general, a topology is taken as reliable if tree reconstruction results in the same topology for at least 95% of the datasets generated by bootstrapping. This is a quite strict approach and it has been shown that subtrees of a tree may be accepted as being significant if they are supported by only 70% of the trees [66]. Using the PHYLIP package, bootstrap datasets were generated with seqboot and used as input data for neighbor. PHYML also offers an option that allows a bootstrap analysis of the original data. This results in a set of trees which can be visualized as a *consensus network *using SplitsTree4 [67]. The specification of a cutoff value allows a clearer view of the bootstrap tree/network where only those edges which are supported by boostrap values higher than the cutoff are included.

### Detection of sequence motifs and their representation

The program meme [68,69] was used to detect the sequence motifs in C domains. Meme discovers one or more motifs in a collection of unaligned DNA or protein sequences. The C domain subtypes were aligned using MUSCLE [52,53], the multiple alignments were visualized using JalView [70] and the motifs found by meme were extracted (cut out). It was ascertained that the C domain motifs described by Sieber and Marahiel [2] were included as well as remarkable sequence positions in the proximity of the motifs, such as single conserved residues or positions which were important for discerning the subtypes. The dissected motif sequences were used to create pHHMs with HMMER and also to create sequence logos using seqlogo by Crooks *et al*. [31]. Sequence logos were prefered over consensus sequences, as they provide a more precise description of sequence similarity and reveal significant features of the alignment which are otherwise difficult to perceive. For sequence logos, positions with > 10% gaps were removed. Sequence logos of all C domain motifs created with seqlogo are available online as Additional file 9.

## Authors' contributions

Authors CR and IH are the principle authors of this article. IH gathered the sequences and constructed and analyzed the phylogenetic trees. CR analyzed the subtype determining residues and constructed and interpreted the sequence logos. CR wrote the manuscript with the participation of IH in several sections. Authors TW and WW made important contributions to biological questions and DHH contributed to phylogenetic questions. All authors read and approved the final version of the manuscript.

## Supplementary Material

Additional file 1 **Phylogenetic tree of all 525 C domain sequences of this study reconstructed using phyml**. Zipped Nexus file (file name extension .nex.zip, to be unpacked and opened with SplitsTree [67,71]). Click here for file

Additional file 2 **Comparison of the logos generated from the pHMMs for the 3 subtypes ^L^C_L_, Starter and ^D^C_L _domain using LogoMat-P **[72]. Click here for file

Additional file 3 Phylogenetic tree of all 525 C domain sequences of this study reconstructed using phyml. Click here for file

Additional file 4 **HMMER outputs of glyco-NRPS: fossils in ComC and ComD**. ZIP file containing two text files. Click here for file

Additional file 5 **Profile HMMs of the 4 complete C domain subtypes (^L^C_L_, Starter, ^D^C_L_, Dual) which can be used to detect and distinguish between the subtypes**. Zipped text file (file name extension .hmm to be used with the program package HMMER [49]). Click here for file

Additional file 6 **Aligned full length condensation domains of this study**. Zipped sequence file (aligned protein sequences in FASTA format). Click here for file

Additional file 7 **Profile HMMs of all 7 motifs of all subtypes (^L^C_L_, Starter, ^D^C_L_, Dual)**. Zipped text file (file name extension .hmm to be used with the program package HMMER [49]. Click here for file

Additional file 8 Listing of NRPSs from known biosynthesis clusters used in this study. Click here for file

Additional file 9 **Sequence logos of all C domain motifs created with weblogo **[31]. ZIP file containing image files in the PNG file format. Click here for file
