# Supplementary figures and images for: Phylogenetic analysis of condensation domains in NRPS sheds light on their functional evolution
Source: BMC Evol Biol. 2007 May 16;7:78. doi: 10.1186/1471-2148-7-78 (PMC1894796; doi:10.1186/1471-2148-7-78)

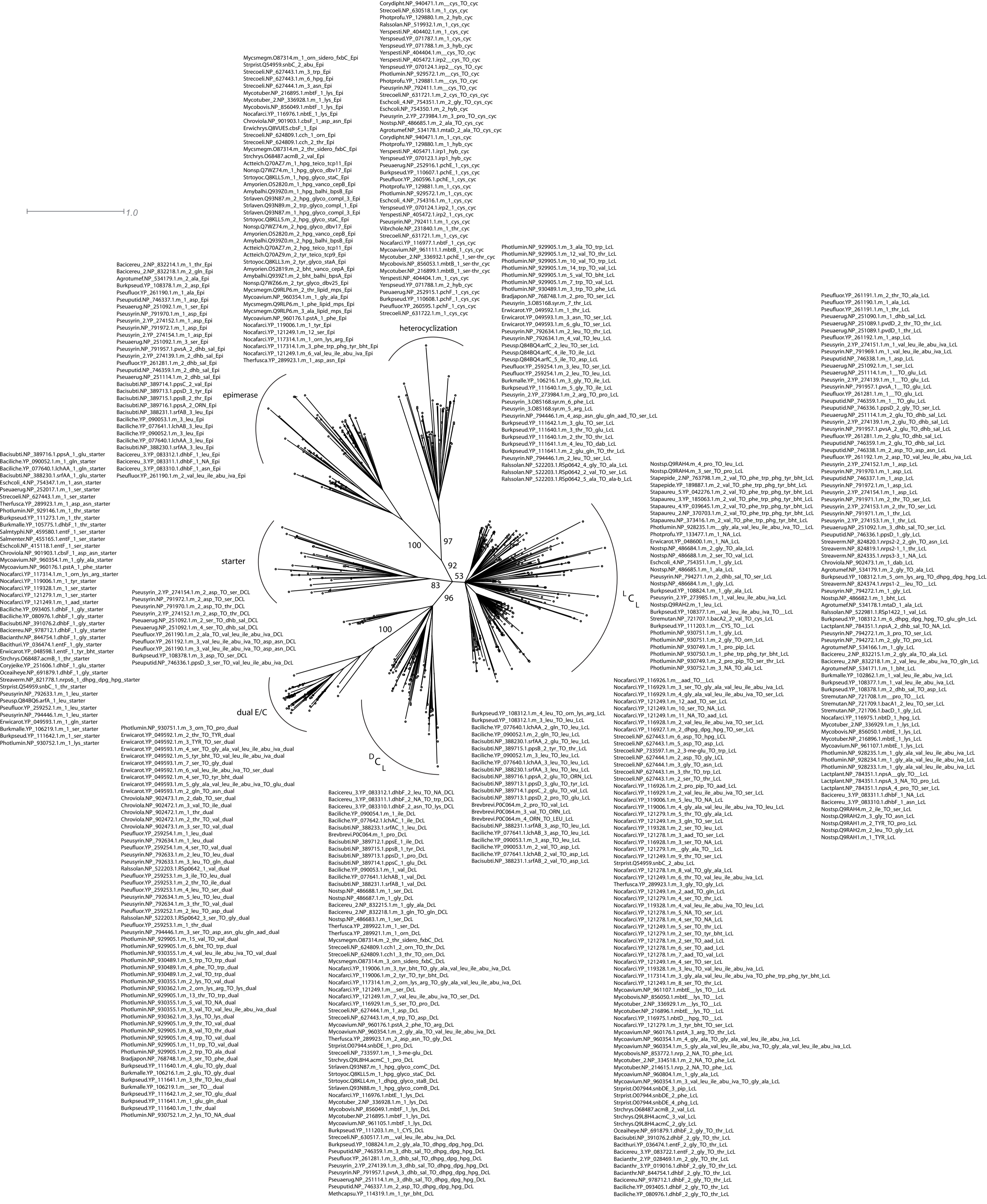

Supplement: Additional file 2 — Comparison of the logos generated from the pHMMs for the 3 subtypes LCL, Starter and DCL domain using LogoMat-P [72]. [file 1471-2148-7-78-S2.pdf]

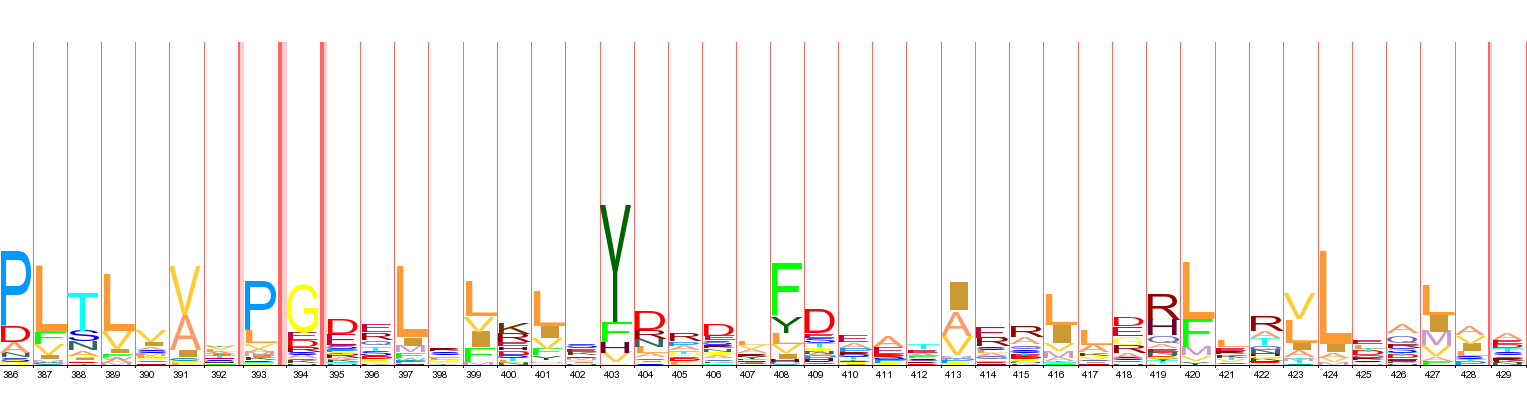

Supplement: Additional file 9 — Sequence logos of all C domain motifs created with weblogo [31]. ZIP file containing image files in the PNG file format. [file 1471-2148-7-78-S9.zip › logosDCL/DCL_386-429.png]

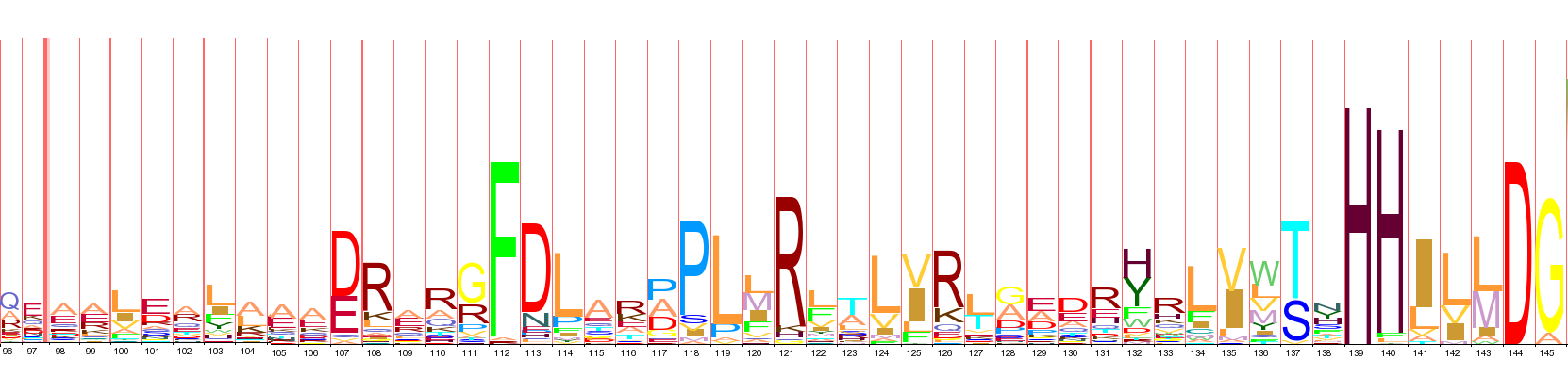

Supplement: Additional file 9 — Sequence logos of all C domain motifs created with weblogo [31]. ZIP file containing image files in the PNG file format. [file 1471-2148-7-78-S9.zip › logosDCL/DCL_96-145.png]

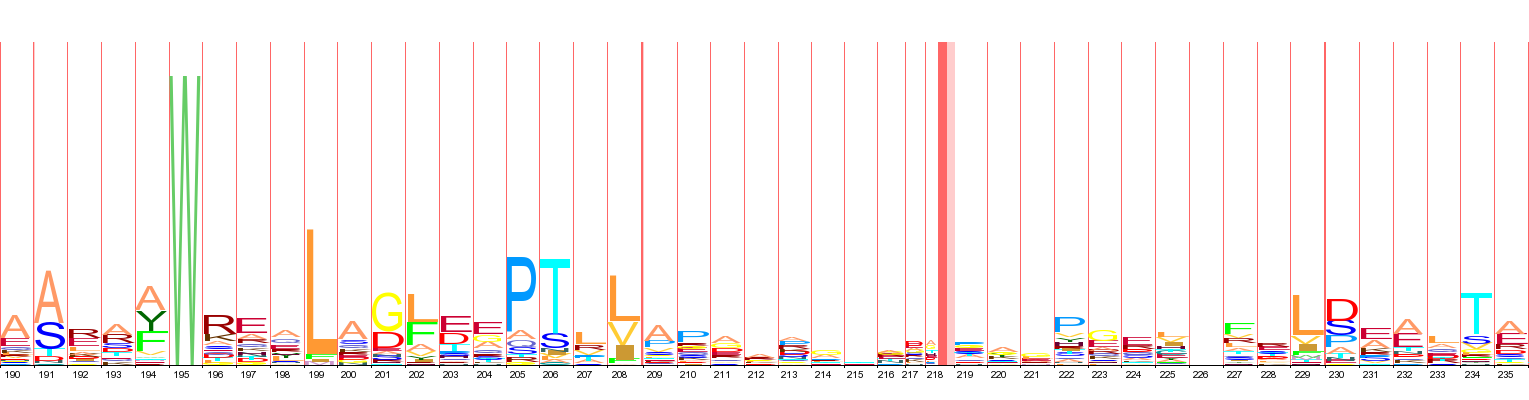

Supplement: Additional file 9 — Sequence logos of all C domain motifs created with weblogo [31]. ZIP file containing image files in the PNG file format. [file 1471-2148-7-78-S9.zip › logosDCL/DCL_190-235.png]

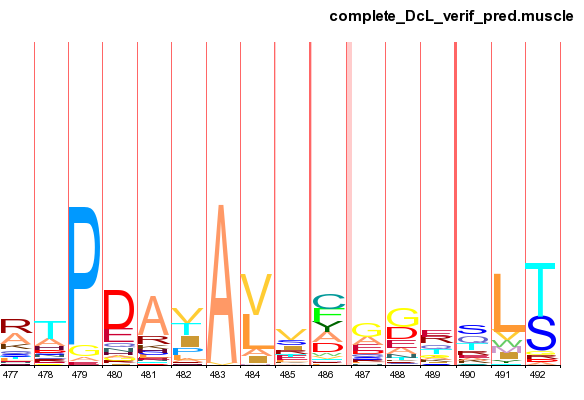

Supplement: Additional file 9 — Sequence logos of all C domain motifs created with weblogo [31]. ZIP file containing image files in the PNG file format. [file 1471-2148-7-78-S9.zip › logosDCL/DCL_477-492.png]

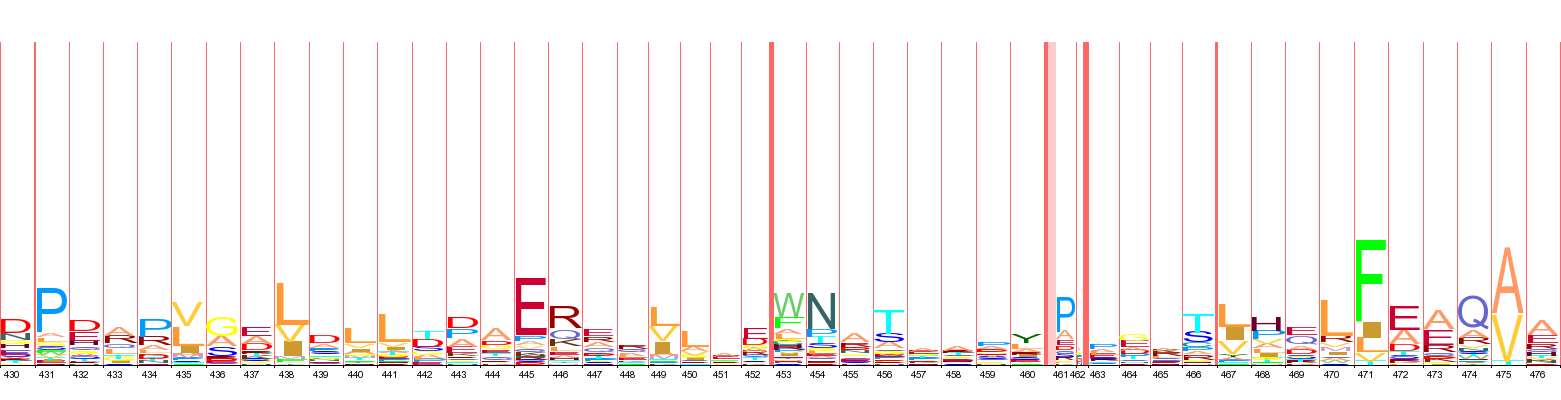

Supplement: Additional file 9 — Sequence logos of all C domain motifs created with weblogo [31]. ZIP file containing image files in the PNG file format. [file 1471-2148-7-78-S9.zip › logosDCL/DCL_430-476.png]

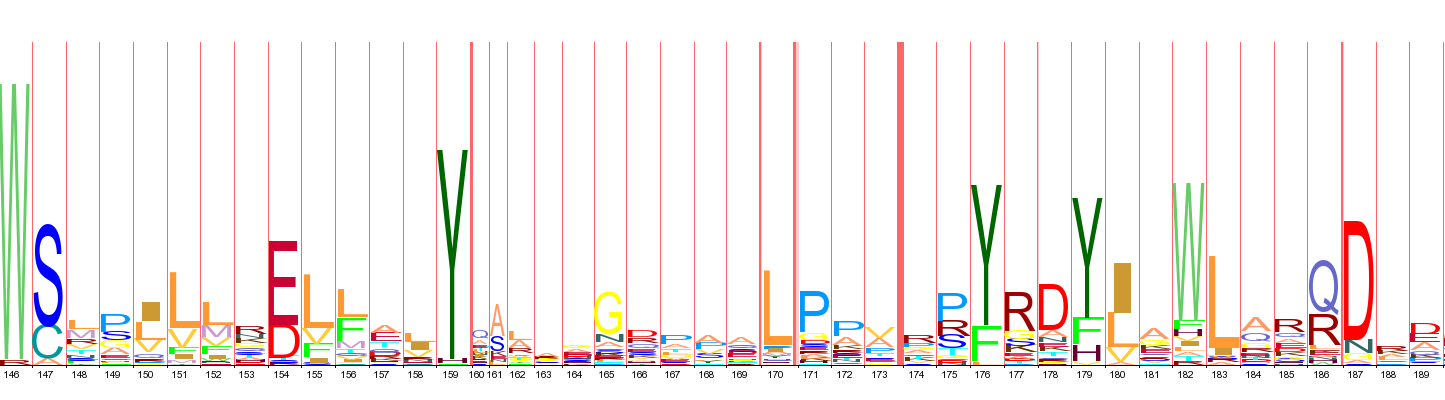

Supplement: Additional file 9 — Sequence logos of all C domain motifs created with weblogo [31]. ZIP file containing image files in the PNG file format. [file 1471-2148-7-78-S9.zip › logosDCL/DCL_146-189.png]

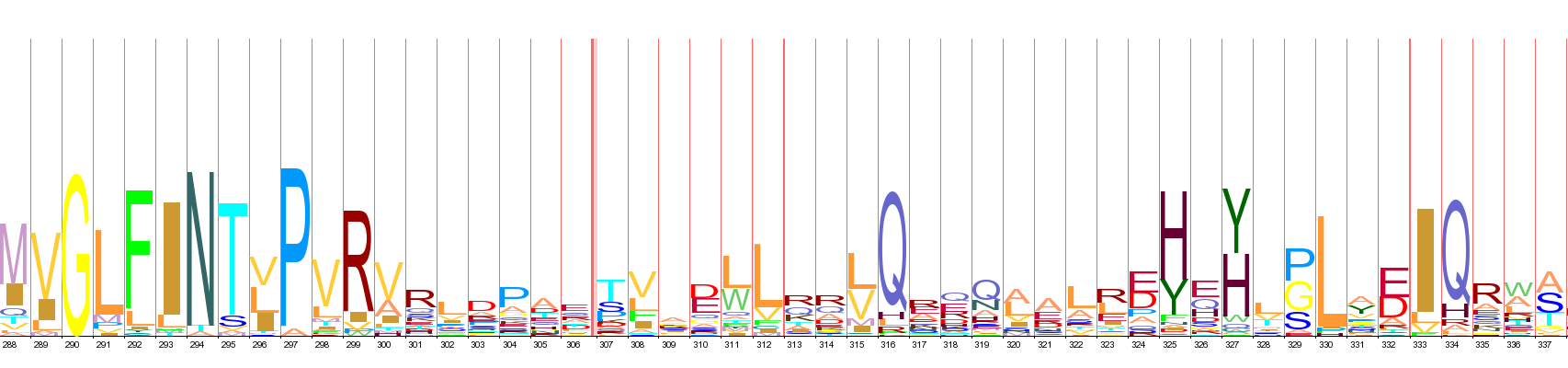

Supplement: Additional file 9 — Sequence logos of all C domain motifs created with weblogo [31]. ZIP file containing image files in the PNG file format. [file 1471-2148-7-78-S9.zip › logosDCL/DCL_288-337.png]

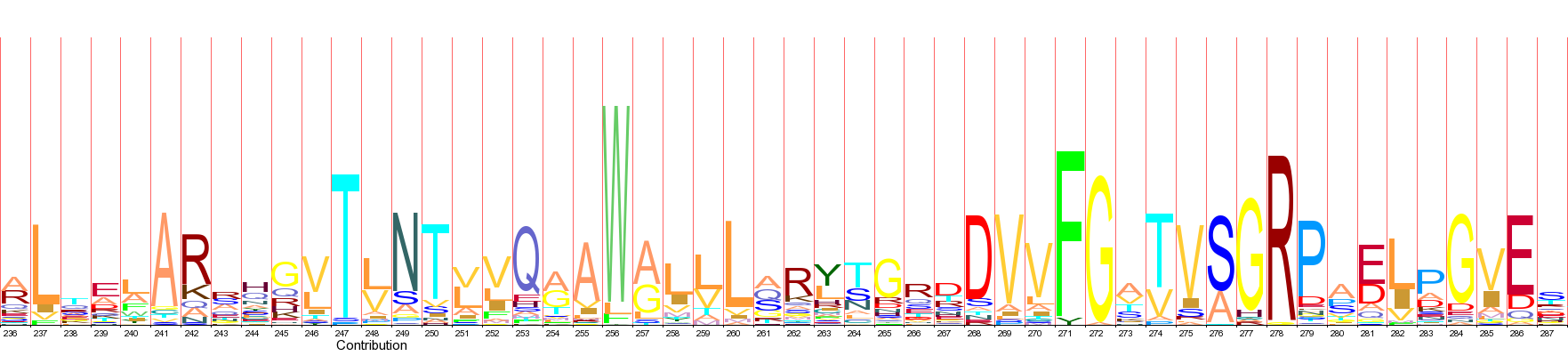

Supplement: Additional file 9 — Sequence logos of all C domain motifs created with weblogo [31]. ZIP file containing image files in the PNG file format. [file 1471-2148-7-78-S9.zip › logosDCL/DCL_236-287.png]

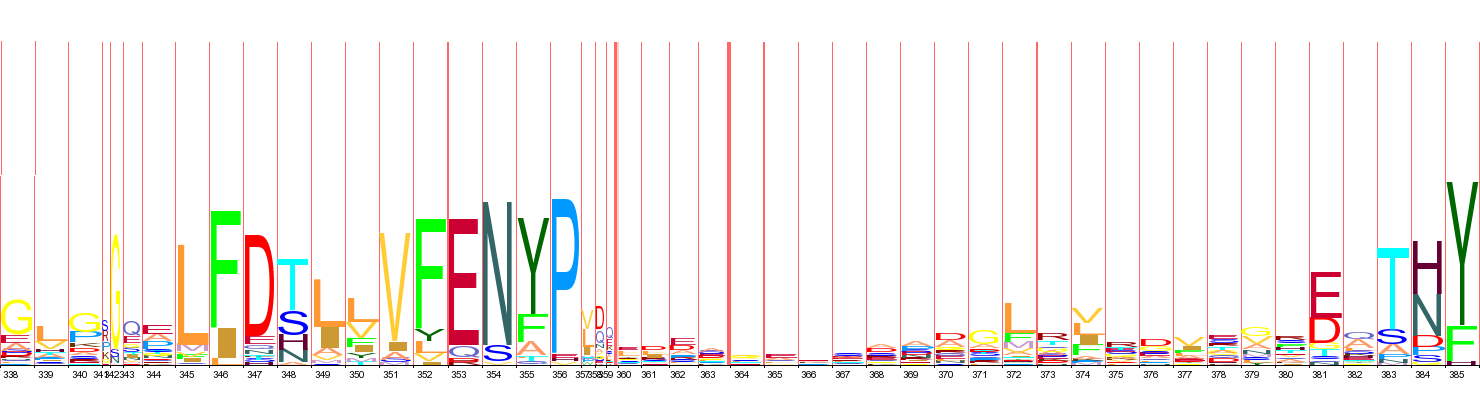

Supplement: Additional file 9 — Sequence logos of all C domain motifs created with weblogo [31]. ZIP file containing image files in the PNG file format. [file 1471-2148-7-78-S9.zip › logosDCL/DCL_338-385.png]

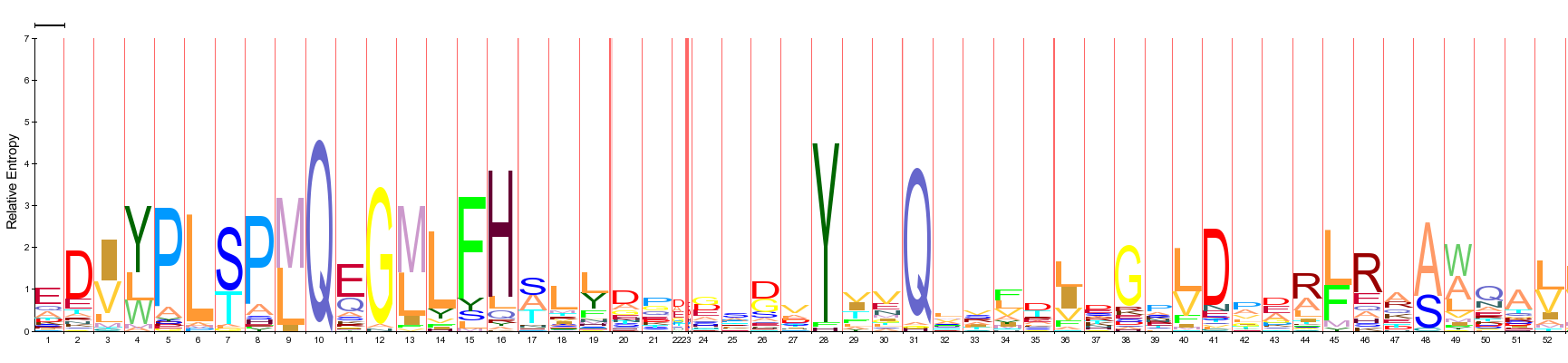

Supplement: Additional file 9 — Sequence logos of all C domain motifs created with weblogo [31]. ZIP file containing image files in the PNG file format. [file 1471-2148-7-78-S9.zip › logosDCL/DCL_1-52.png]

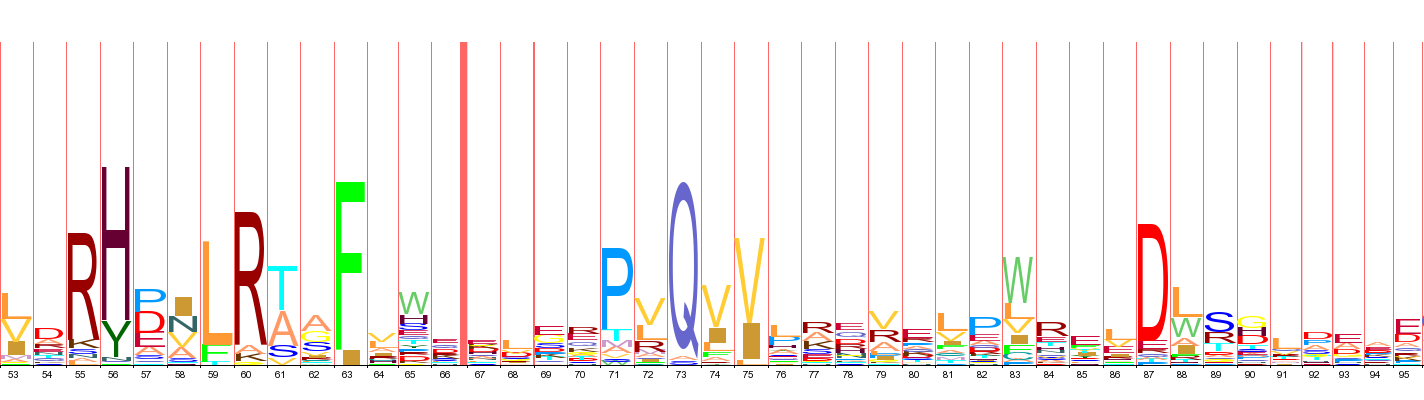

Supplement: Additional file 9 — Sequence logos of all C domain motifs created with weblogo [31]. ZIP file containing image files in the PNG file format. [file 1471-2148-7-78-S9.zip › logosDCL/DCL_53-95.png]

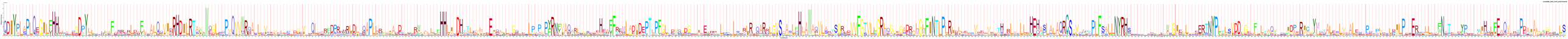

Supplement: Additional file 9 — Sequence logos of all C domain motifs created with weblogo [31]. ZIP file containing image files in the PNG file format. [file 1471-2148-7-78-S9.zip › logosDual/dual_complete.png]

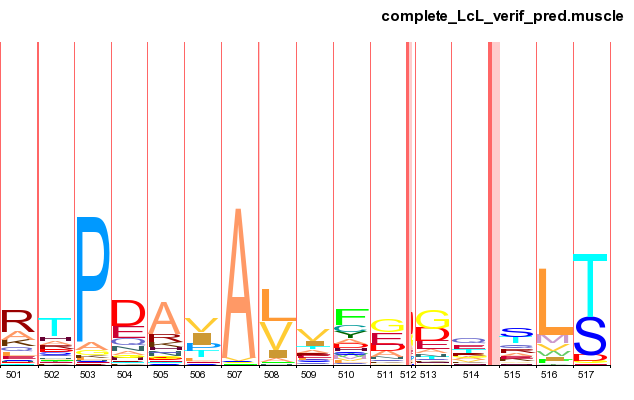

Supplement: Additional file 9 — Sequence logos of all C domain motifs created with weblogo [31]. ZIP file containing image files in the PNG file format. [file 1471-2148-7-78-S9.zip › logosLCL/LCL_501-517.png]

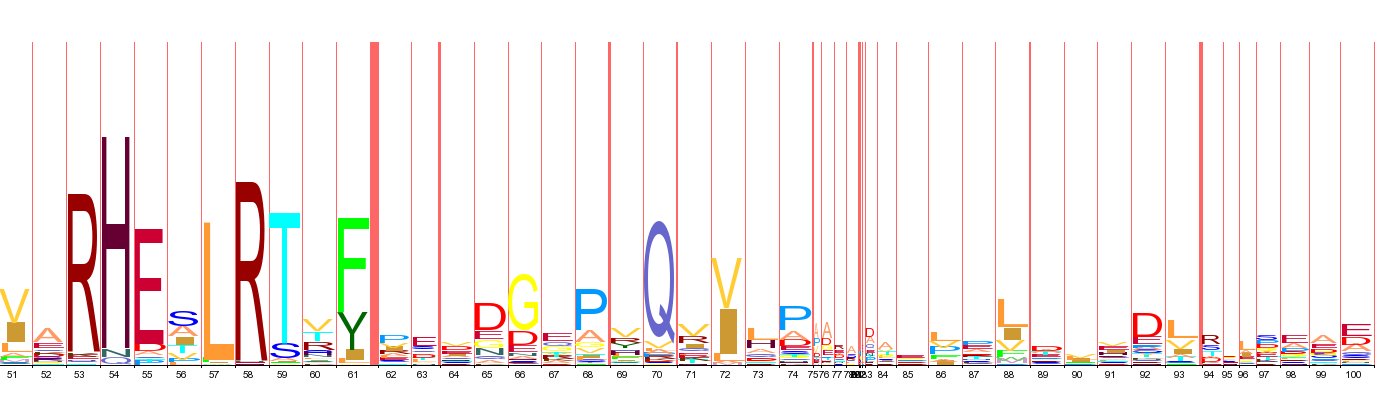

Supplement: Additional file 9 — Sequence logos of all C domain motifs created with weblogo [31]. ZIP file containing image files in the PNG file format. [file 1471-2148-7-78-S9.zip › logosLCL/LCL_51-100.png]

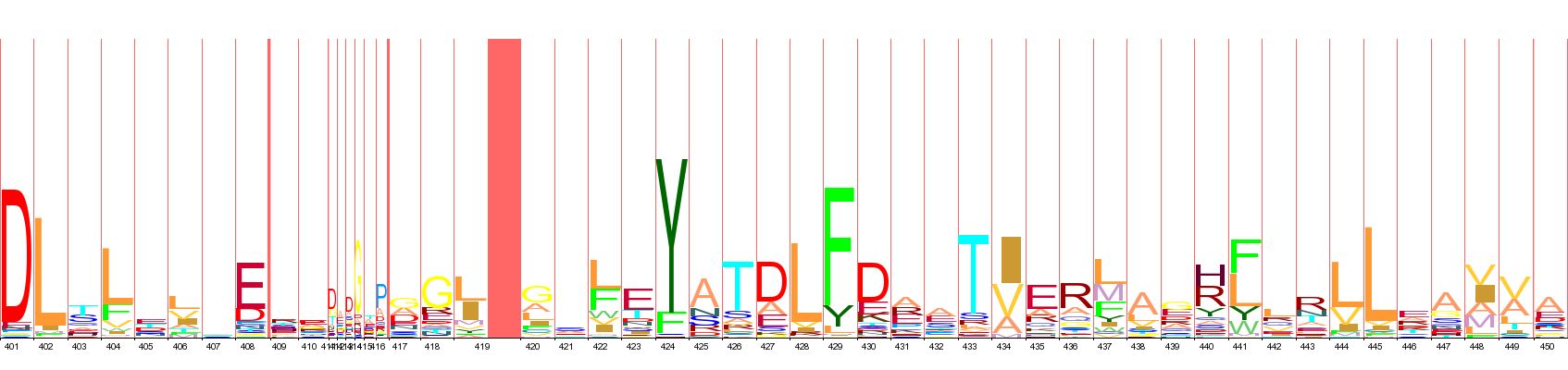

Supplement: Additional file 9 — Sequence logos of all C domain motifs created with weblogo [31]. ZIP file containing image files in the PNG file format. [file 1471-2148-7-78-S9.zip › logosLCL/LCL_401-450.png]

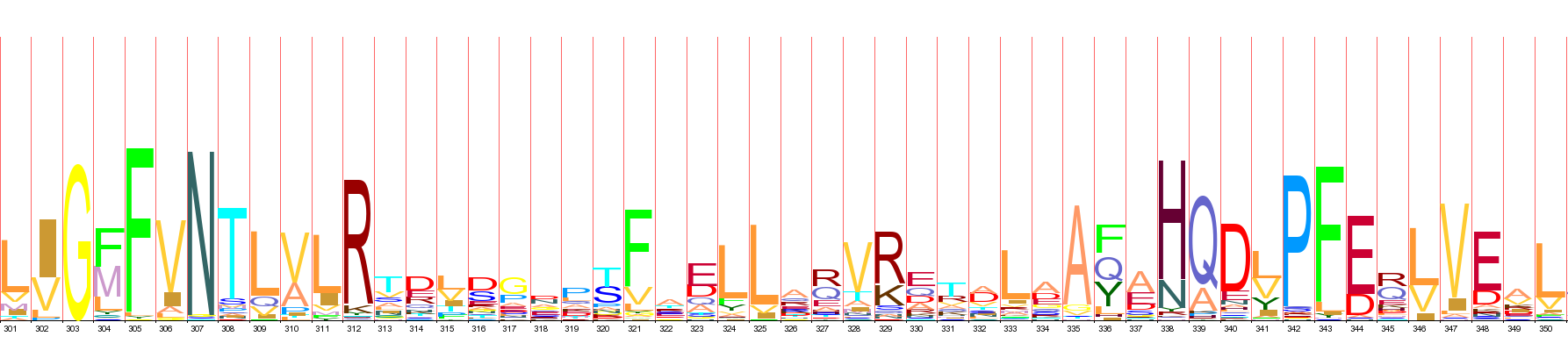

Supplement: Additional file 9 — Sequence logos of all C domain motifs created with weblogo [31]. ZIP file containing image files in the PNG file format. [file 1471-2148-7-78-S9.zip › logosLCL/LCL_301-350.png]

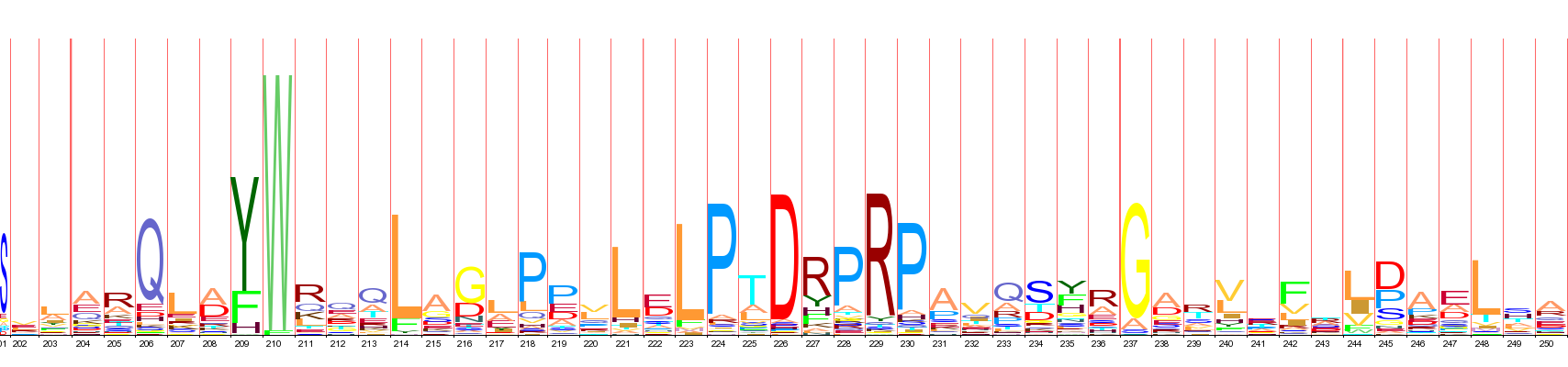

Supplement: Additional file 9 — Sequence logos of all C domain motifs created with weblogo [31]. ZIP file containing image files in the PNG file format. [file 1471-2148-7-78-S9.zip › logosLCL/LCL_201-250.png]

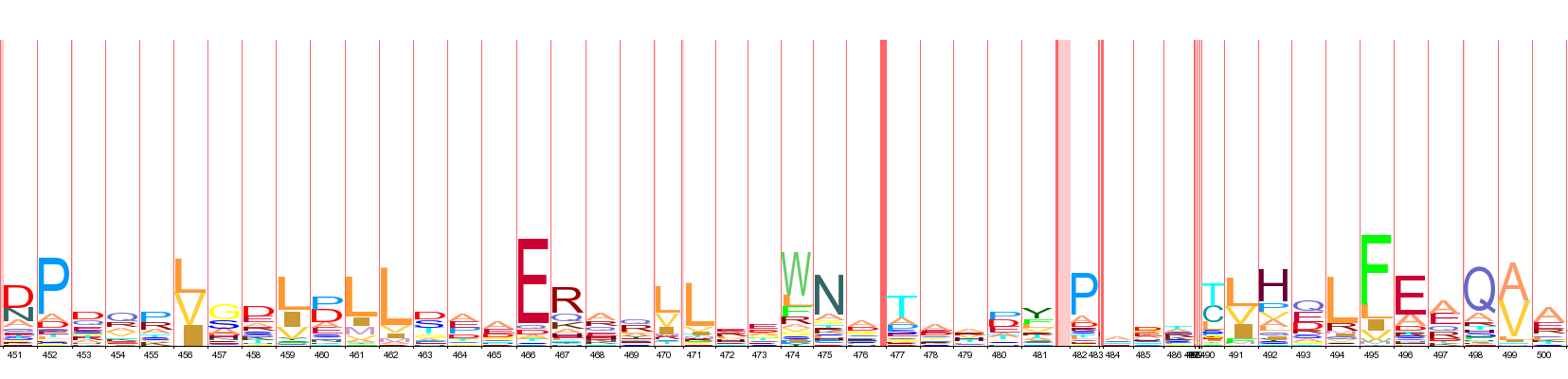

Supplement: Additional file 9 — Sequence logos of all C domain motifs created with weblogo [31]. ZIP file containing image files in the PNG file format. [file 1471-2148-7-78-S9.zip › logosLCL/LCL_451-500.png]

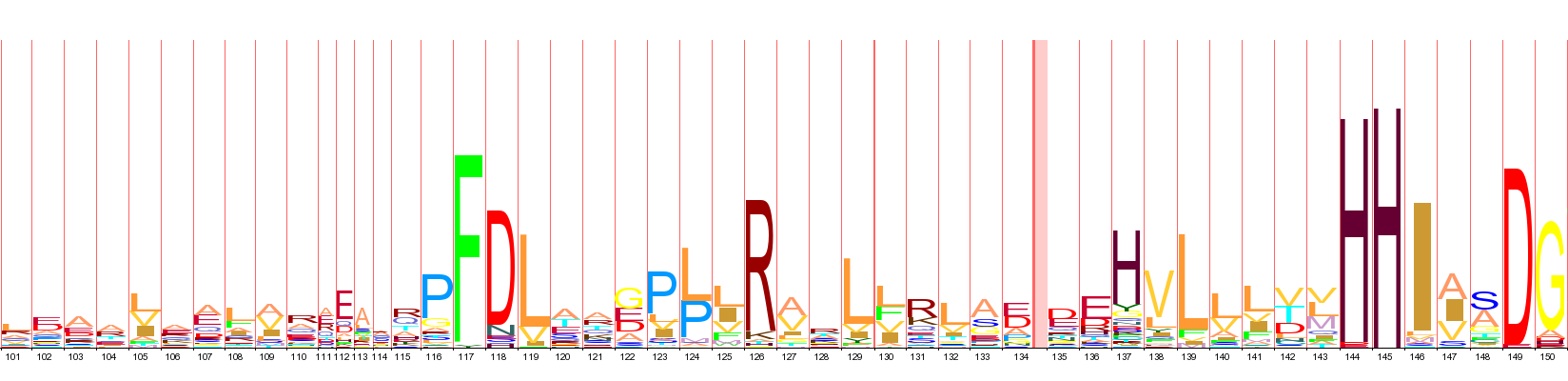

Supplement: Additional file 9 — Sequence logos of all C domain motifs created with weblogo [31]. ZIP file containing image files in the PNG file format. [file 1471-2148-7-78-S9.zip › logosLCL/LCL_101-150.png]

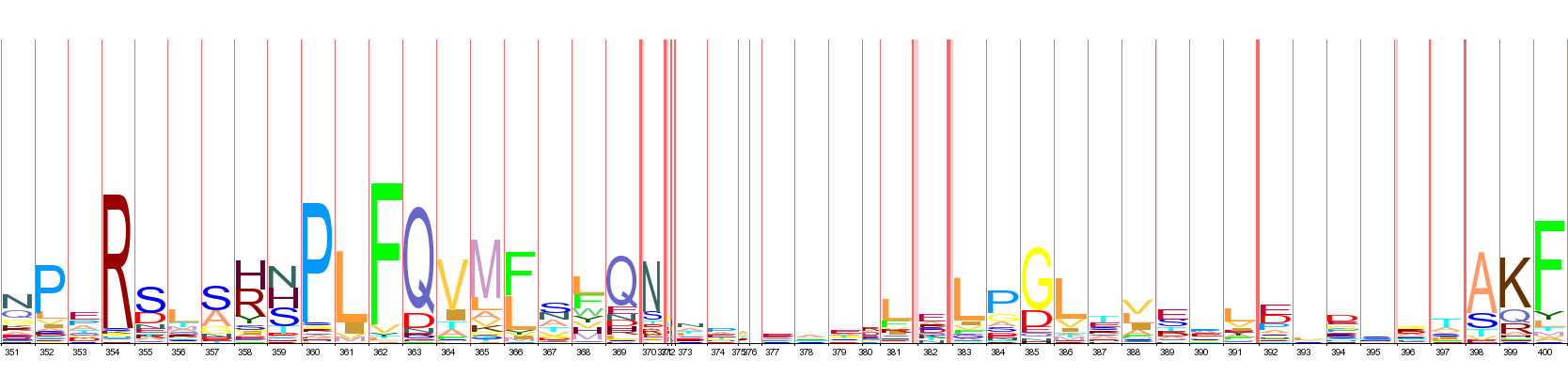

Supplement: Additional file 9 — Sequence logos of all C domain motifs created with weblogo [31]. ZIP file containing image files in the PNG file format. [file 1471-2148-7-78-S9.zip › logosLCL/LCL_351-400.png]

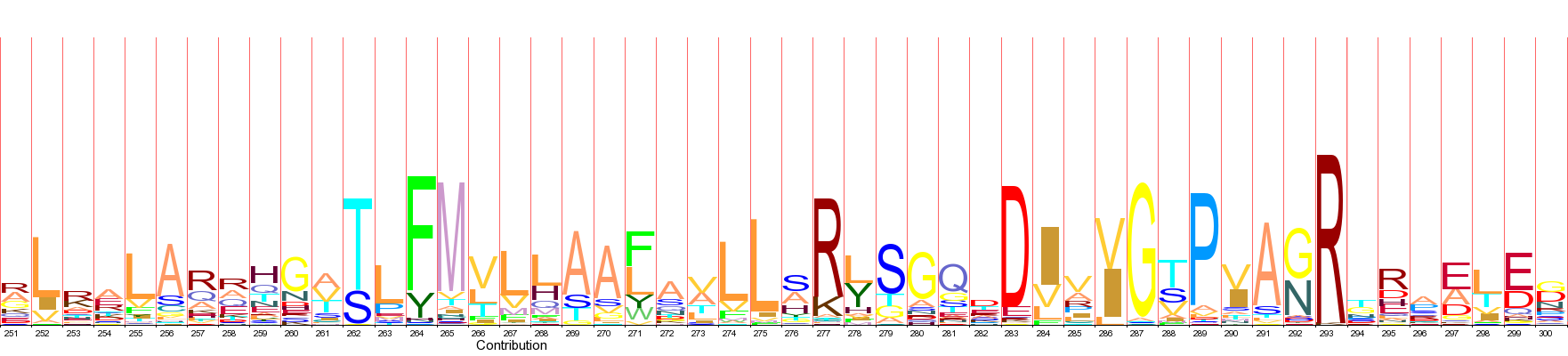

Supplement: Additional file 9 — Sequence logos of all C domain motifs created with weblogo [31]. ZIP file containing image files in the PNG file format. [file 1471-2148-7-78-S9.zip › logosLCL/LCL_251-300.png]

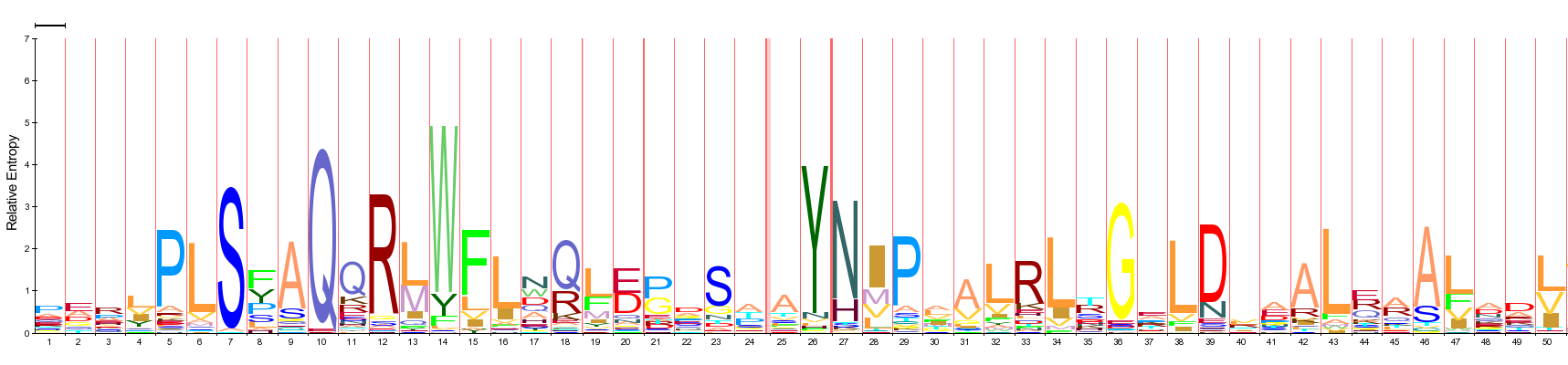

Supplement: Additional file 9 — Sequence logos of all C domain motifs created with weblogo [31]. ZIP file containing image files in the PNG file format. [file 1471-2148-7-78-S9.zip › logosLCL/LCL_1-50.png]

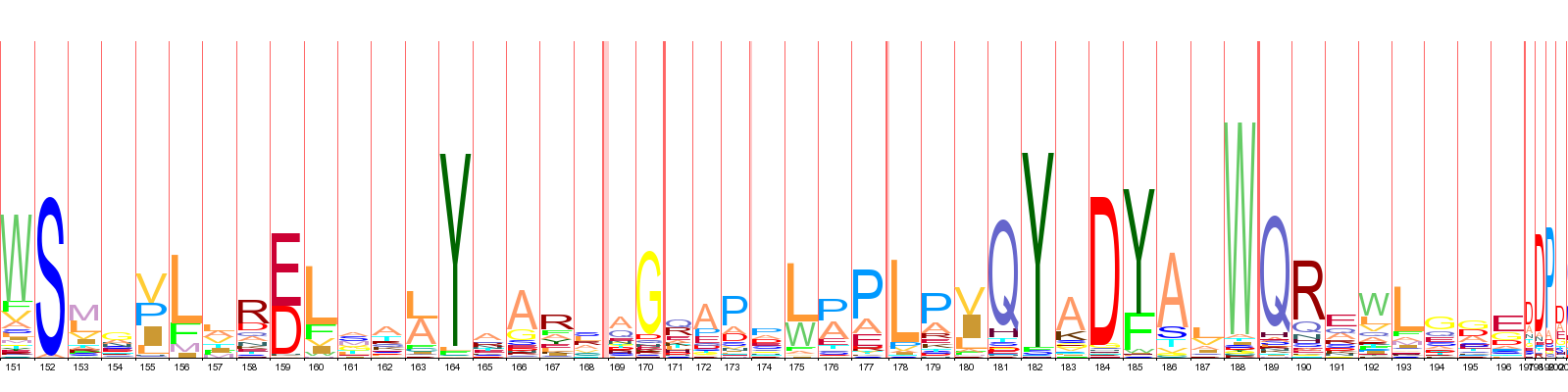

Supplement: Additional file 9 — Sequence logos of all C domain motifs created with weblogo [31]. ZIP file containing image files in the PNG file format. [file 1471-2148-7-78-S9.zip › logosLCL/LCL_151-200.png]

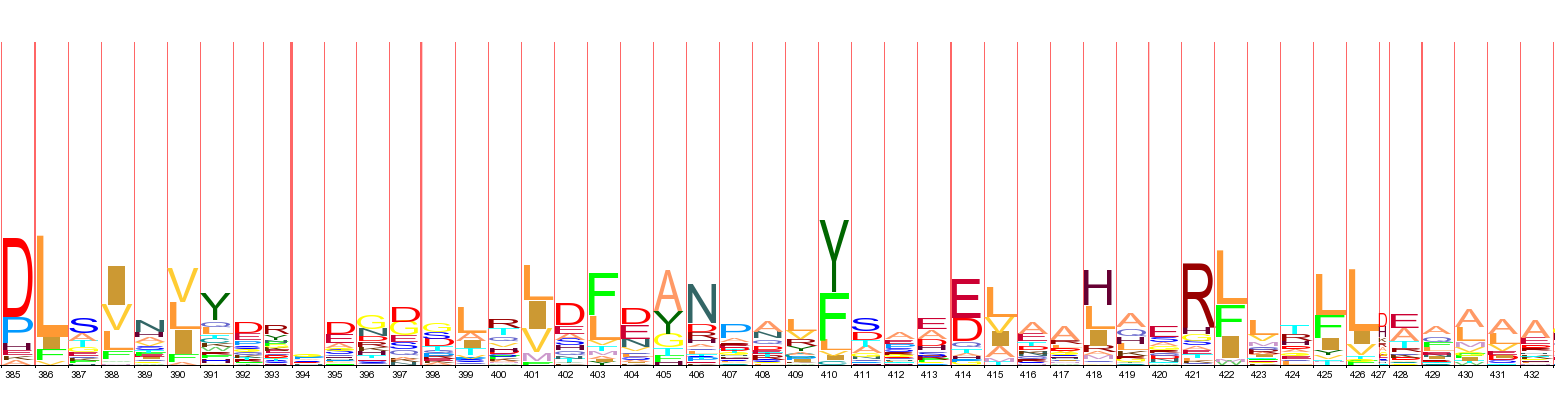

Supplement: Additional file 9 — Sequence logos of all C domain motifs created with weblogo [31]. ZIP file containing image files in the PNG file format. [file 1471-2148-7-78-S9.zip › logosStarter/Starter_385-432.png]

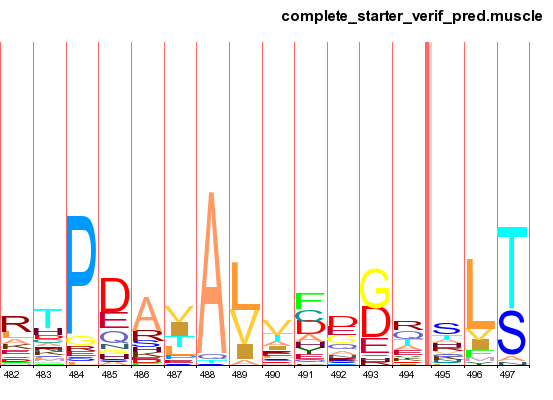

Supplement: Additional file 9 — Sequence logos of all C domain motifs created with weblogo [31]. ZIP file containing image files in the PNG file format. [file 1471-2148-7-78-S9.zip › logosStarter/Starter_482-497.png]

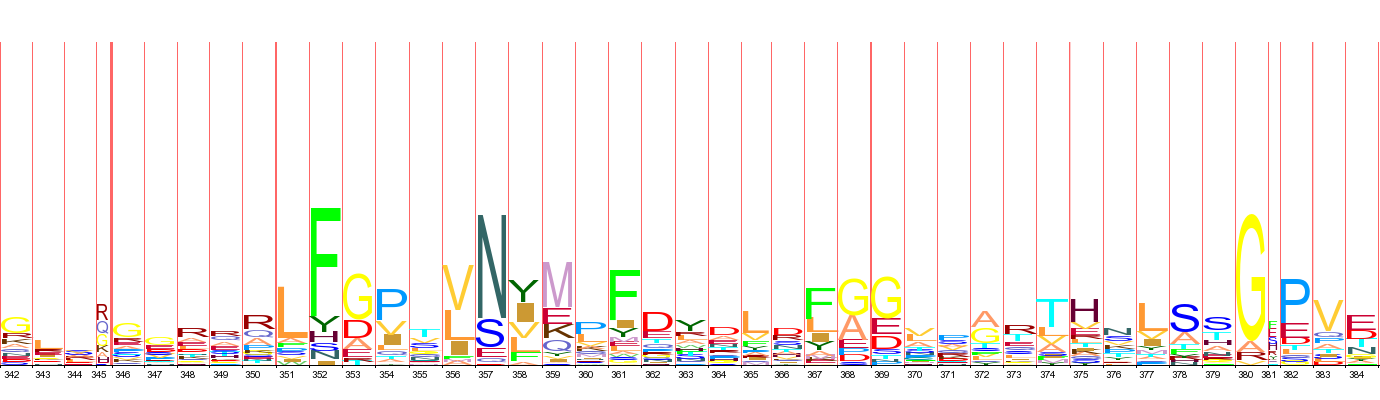

Supplement: Additional file 9 — Sequence logos of all C domain motifs created with weblogo [31]. ZIP file containing image files in the PNG file format. [file 1471-2148-7-78-S9.zip › logosStarter/Starter_342-384.png]

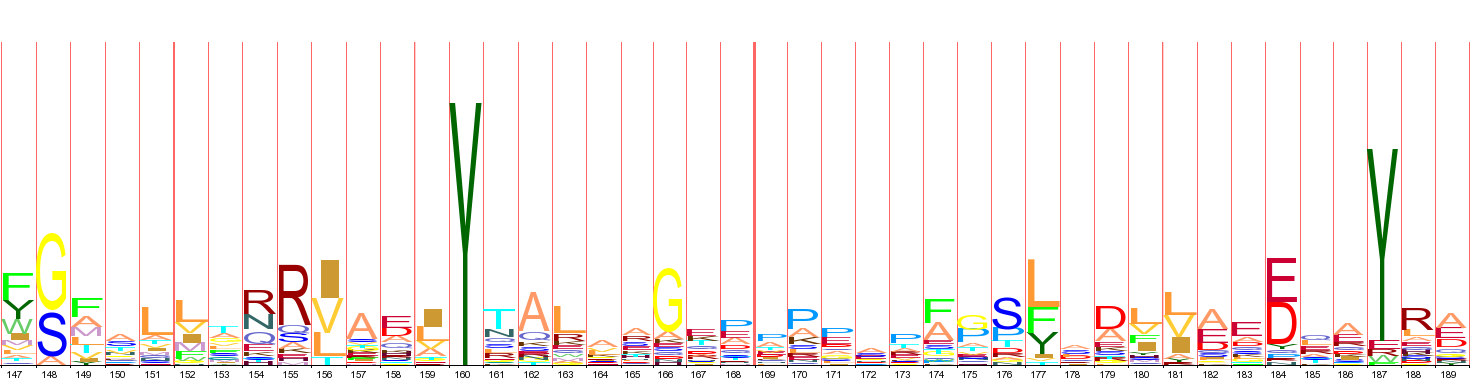

Supplement: Additional file 9 — Sequence logos of all C domain motifs created with weblogo [31]. ZIP file containing image files in the PNG file format. [file 1471-2148-7-78-S9.zip › logosStarter/Starter_147-189.png]

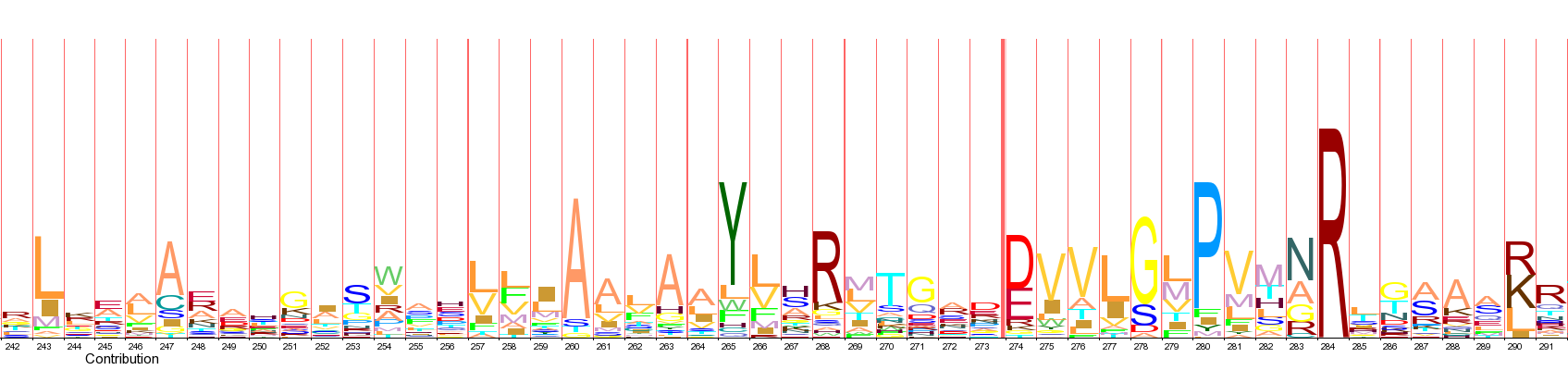

Supplement: Additional file 9 — Sequence logos of all C domain motifs created with weblogo [31]. ZIP file containing image files in the PNG file format. [file 1471-2148-7-78-S9.zip › logosStarter/Starter_242-291.png]

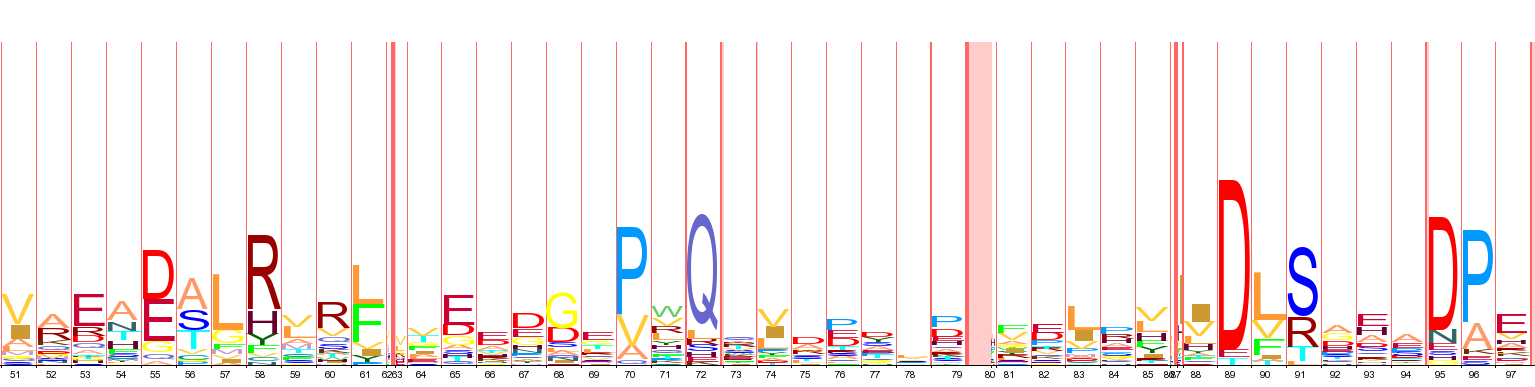

Supplement: Additional file 9 — Sequence logos of all C domain motifs created with weblogo [31]. ZIP file containing image files in the PNG file format. [file 1471-2148-7-78-S9.zip › logosStarter/Starter_51-97.png]

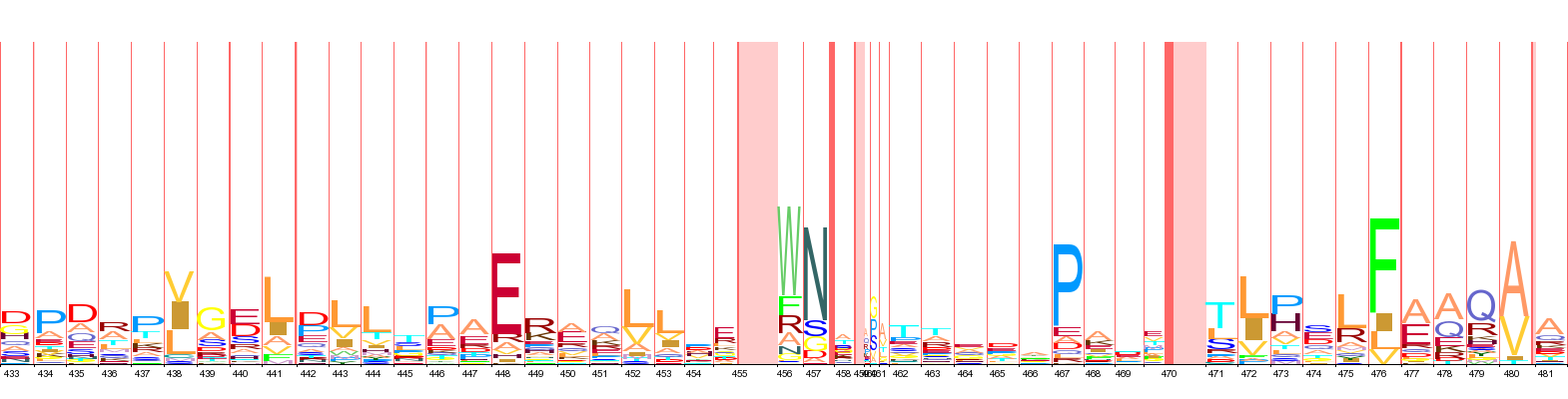

Supplement: Additional file 9 — Sequence logos of all C domain motifs created with weblogo [31]. ZIP file containing image files in the PNG file format. [file 1471-2148-7-78-S9.zip › logosStarter/Starter_433-481.png]

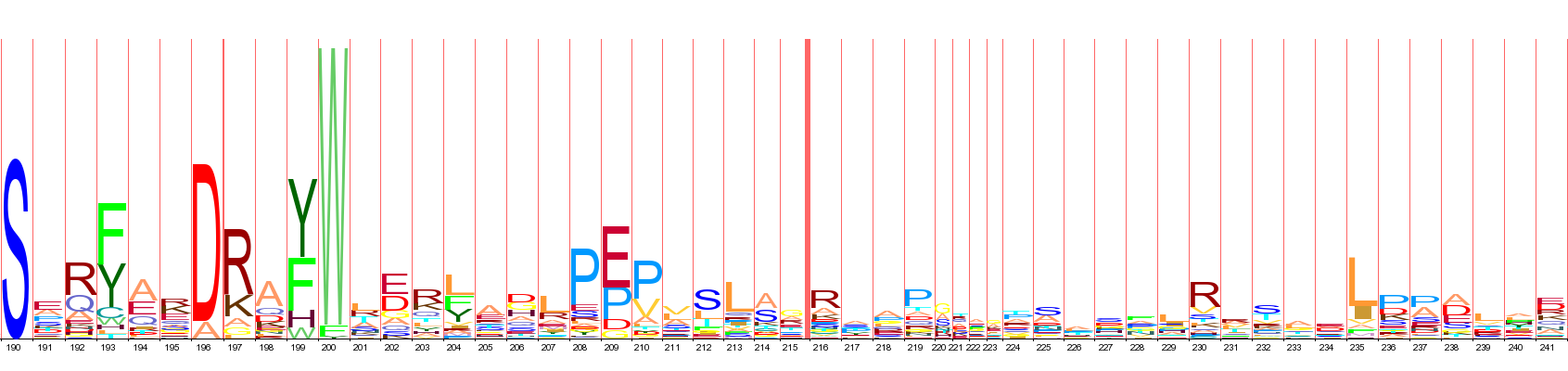

Supplement: Additional file 9 — Sequence logos of all C domain motifs created with weblogo [31]. ZIP file containing image files in the PNG file format. [file 1471-2148-7-78-S9.zip › logosStarter/Starter_190-241.png]

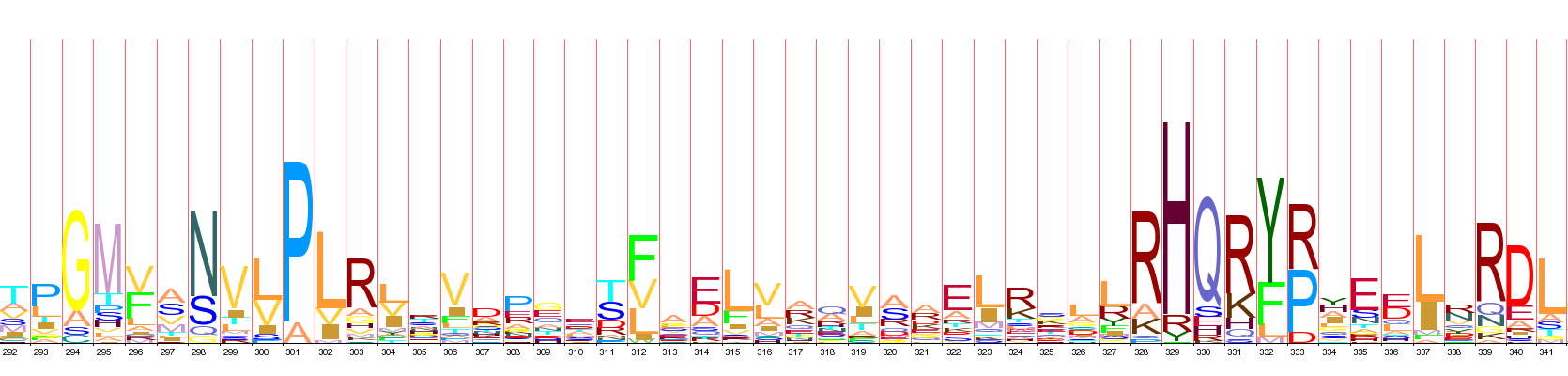

Supplement: Additional file 9 — Sequence logos of all C domain motifs created with weblogo [31]. ZIP file containing image files in the PNG file format. [file 1471-2148-7-78-S9.zip › logosStarter/Starter_292-341.png]

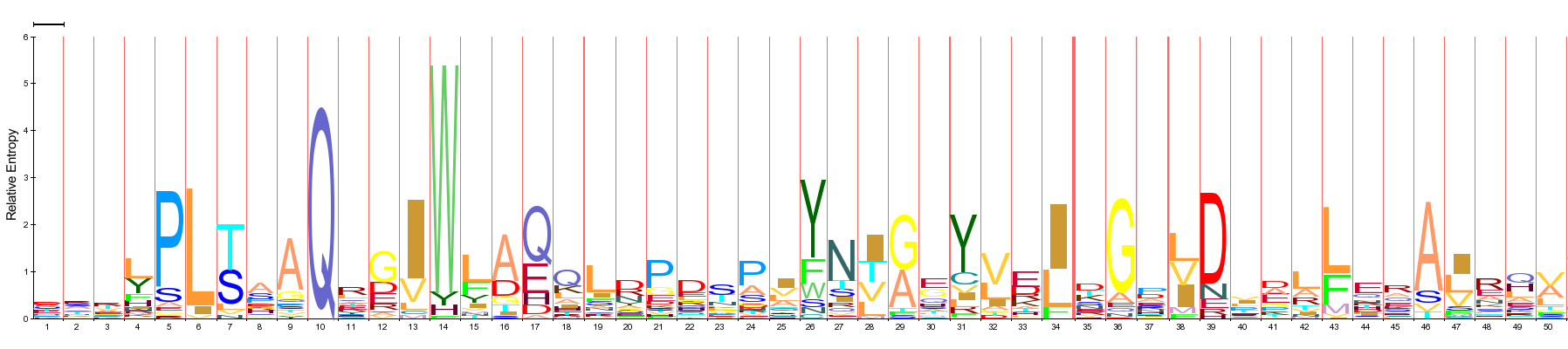

Supplement: Additional file 9 — Sequence logos of all C domain motifs created with weblogo [31]. ZIP file containing image files in the PNG file format. [file 1471-2148-7-78-S9.zip › logosStarter/Starter_1-50.png]

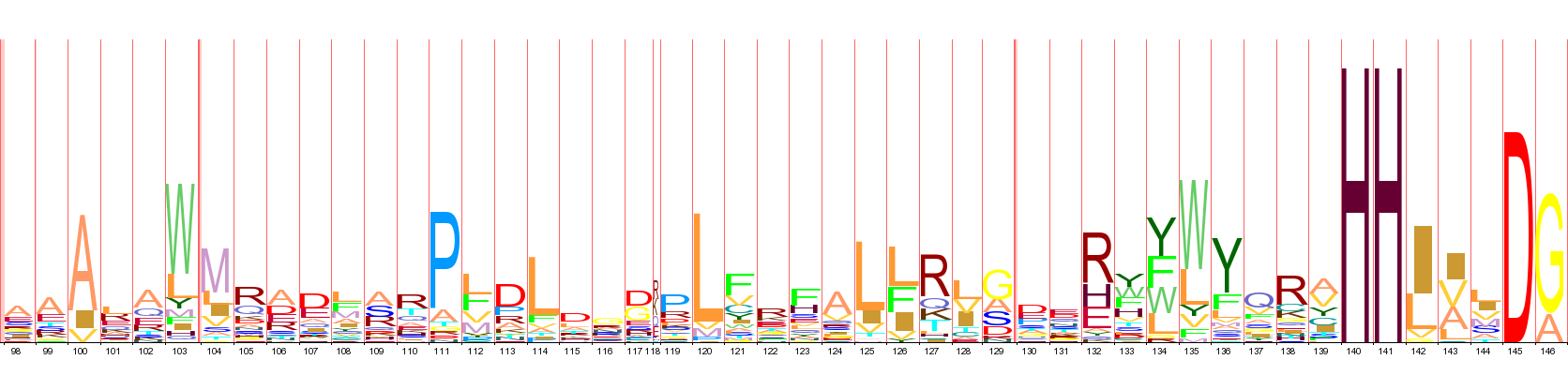

Supplement: Additional file 9 — Sequence logos of all C domain motifs created with weblogo [31]. ZIP file containing image files in the PNG file format. [file 1471-2148-7-78-S9.zip › logosStarter/Starter_98-146.png]
