# Supplementary material for: Phylogenetic analysis of condensation domains in NRPS sheds light on their functional evolution
Source: BMC Evol Biol. 2007 May 16;7:78. doi: 10.1186/1471-2148-7-78 (PMC1894796; doi:10.1186/1471-2148-7-78)
Supplement: Additional file 8 — Listing of NRPSs from known biosynthesis clusters used in this study. [file 1471-2148-7-78-S8.pdf]

Listing of the known NRPS biosynthetic clusters used in the study described in the article: Phylogenetic Analysis of Condensation Domains in NRPS Sheds Light on Their Functional Evolution

Christian Rausch, Ilka Hoof, Tilmann Weber, Wolfgang Wohlleben and Daniel H. Huson

December 14, 2006

**Organism**	**UniProt ID**	**Module**	**Domain structure**	**Substrate**
Actinoplanes teichomyceticus				
	Q70AZ9	tcp9_1	A-T	hpg
		tcp9_2	C-A-T-E	tyr
	Q70AZ8	tcp10	C-A-T	dhpg
	Q70AZ7	tcp11_1	C-A-T-E	hpg
		tcp11_2	C-A-T-E	hpg
		tcp11_3	C-A-T	bht
	Q70AZ6	tcp12	C-A-T-X*-Te	dhpg
Amycolatopsis balhimycina				
	Q939Z1	bpsA_1	A-T	leu
		bpsA_2	C-A-T-E	bht
		bpsA_3	C-A-T	asn
	Q939Z0	bpsB_1	C-A-T-E	hpg
		bpsB_2	C-A-T-E	hpg
		bpsB_3	C-A-T	bht
	Q939Y9	bpsC	C-A-T-X*-Te	dhpg
Amycolatopsis orientalis				
	O52819	cepA_1	A-T	leu
		cepA_2	C-A-T-E	bht
		cepA_3	C-A-T	asn
	O52820	cepB_1	C-A-T-E	hpg
		cepB_2	C-A-T-E	hpg
		cepB_3	C-A-T	bht
	O52821	cepC	C-A-T-X*-Te	dhpg
Mycobacterium bovis				
	Q7TYQ8	mbtF	C-A-T-E	lys
	Q7TYQ4	mbtB	C-A-T-Te	ser-thr
Mycobacterium smegmatis				
	O87313	fxbB_1	A-T-E	orn
		fxbB_2	C-A-T	ala-b

		fxbB_3	C	
	O87314	fxbC_1	A-T-E	orn
		fxbC_2	C-A-T-E	thr
		fxbC_3	C-A-T	orn
		fxbC_4	C-A-T-Te	ser_thr_ser-thr_dht_dhpg_dpg_hpg
Nocardia lactamdurans				
	P27743	pcbAB_1	A-T	aad
		pcbAB_2	C-A-T	cys
		pcbAB_3	C-A-T-E-Te	val
Nonomuraea sp. ATCC 39727				
	Q7WZ66	dbv25_1	A-T	hpg
		dbv25_2	C-A-T-E	tyr
	Q7WZ65	dbv26	C-A-T	dpg
	Q7WZ74	dbv17_1	C-A-T-E	hpg
		dbv17_2	C-A-T-E	hpg
		dbv17_3	C-A-T	tyr
	Q7WZ75	dbv16	C-A-T-X*-Te	dhpg
Pseudomonas syringae pv. syringae				
	O85168	syr_1	A-T	ser
		syr_2	C-A-T	ser
		syr_3	C-A-T	dab
		syr_4	C-A-T	dab
		syr_5	C-A-T	arg
		syr_6	C-A-T	phe
		syr_7	C-A-T	thr
		syr_8	C-A-T	asp
		syr_9	C-T-Te	
Streptomyces chrysomallus				
	Q9S6J9	acmA	A	4-mha
	O68487	acmB_1	C-A-T	thr
		acmB_2	C-A-T-E	val
	Q9L8H4	acmC_1	C-A-T	pro
		acmC_2	C-A-M-T	gly
		acmC_3	C-A-M-T-Te	val
Streptomyces lavendulae				
	Q93N89	comA_1	A-T	hpg
		comA_2	C-A-T-E	trp
	Q93N88	comB	C-A-T-E	hpg
	Q93N87	comC_1	C-A-T-E	hpg
		comC_2	C-A-T-E	hpg
		comC_3	C-A-M-T-E	tyr
	Q93N86	comD	C-A-T-X*-Te	hpg
Streptomyces pristinaespiralis				
	P95819	snbA	A	pip
	Q54959	snbC_1	C-A-T	thr

		snbC_2	C-A-T-E	abu
	O07944	snbDE_1	C-A-T	pro
		snbDE_2	C-A-M-T	phe
		snbDE_3	C-A-T	pip
		snbDE_4	C-A-T-Te	phg
Streptomyces toyocaensis				
	Q8KLL3	staA_1	A-T	hpg
		staA_2	C-A-T-E	tyr
	Q8KLL4	staB	C-A-T-E	dhpg
	Q8KLL5	staC_1	C-A-T-E	hpg
		staC_2	C-A-T-E	hpg-2
		staC_3	C-A-T	bht-3
	Q8KLL6	staD	C-A-T-X*-Te	dhpg
Streptomyces verticillus				
	Q9FB17	blmIII	A-T	cys
	Q9FB26	blmIX	C-A-T	ala
	Q9FB24	blmVII	C-A-T	thr
	Q9FB23	blmVI_0	A-T	NA
		blmVI_1	C-A-T	ser
		blmVI_2	C-A	asn
	Q9FB27	blmX_1	C-A-T	asn
		blmX_2	C-A-T	val_leu_ile_abu_iva
	Q9FB18	blmIV_1	C-A-T	ala-b
		blmIV_2	Cy-A-T	cys
		blmIV_3	Cy	
Streptomyces virginiae				
	O05647	snbDE	A-T-C-A	pro
